# Histone neutralization protects the ischemic brain against the consequences of stroke-associated pneumonia

**DOI:** 10.1007/s00401-026-03097-0

**Published:** 2026-10-09

**Authors:** Dongpei Yin, Anran Li, Ayan Mohamud Yusuf, Olga Shevchuk, Janine Gronewold, Stephanie Thiebes, Tobias Tertel, Chen Wang, Nina Hagemann, Yiqiao Zhang, Claudia Graser, Humeyra Tas, Michael Fleischer, Britta Kaltwasser, Benedikt Frank, Ali Ata Tuz, Vikramjeet Singh, Devon Siemes, Ekaterina Pylaeva, Jadwiga Jablonska, Bente Siebels, Hartmut Schlüter, Jonas Raspe, Michaela Schedel, Yi Liu, Fengyan Jin, Ertugrul Kilic, Bayram Yilmaz, Egor Dzyubenko, Jens Minnerup, Luisa Klotz, Bernd Giebel, Oliver Soehnlein, Matthias Gunzer, Daniel R. Engel, Dirk M. Hermann

**Affiliations:** 1 Department of Neurology, University Hospital Essen, University of Duisburg-Essen, Essen, Germany; 2 Center for Translational and Behavioral Neurosciences, University Hospital Essen, University of Duisburg-Essen, Essen, Germany; 3 Institute of Experimental Immunology and Imaging, University Hospital Essen, University of Duisburg-Essen, Essen, Germany; 4 Institute of Transfusion Medicine, University Hospital Essen, University of Duisburg-Essen, Essen, Germany; 5 Department of Otorhinolaryngology, Head and Neck Surgery, University Hospital Essen, University of Duisburg-Essen, Essen, Germany; 6 German Cancer Consortium (DKTK) Partner Site Düsseldorf/Essen, Essen, Germany; 7 Section Mass Spectrometric Proteomics, Diagnostic Center, University Medical Center Hamburg-Eppendorf, Hamburg, Germany; 8 Translational Pulmonology, Department of Pulmonary Medicine, University Medical Center Essen, Ruhrlandklinik, University of Duisburg-Essen, Essen, Germany; 9 Department of Pulmonary Medicine, University Medical Center Essen, Essen, Germany; 10 Department of Neurology, Central Hospital, Dalian University of Technology, Dalian, Liaoning China; 11 Department of Hematology, First Hospital of Jilin University, Changchun, Jilin China; 12 Department of Physiology, School of Medicine, Istanbul Medeniyet University, Istanbul, Turkey; 13 Izmir International Biomedicine and Genome Institute, Dokuz Eylul University, Izmir, Turkey; 14 Department of Neurology, University of Lübeck and University Hospital Schleswig-Holstein, Lübeck, Germany; 15 German Centre for Cardiovascular Research (DZHK), Partner Site North, Lübeck, Germany; 16 Department of Neurology, University of Münster, Münster, Germany; 17 Department of Experimental Pathology, University Hospital Münster, Münster, Germany; 18 Leibniz-Institut Für Analytische Wissenschaften E.V. (ISAS), Dortmund, Germany

**Keywords:** Ischemic stroke, Neutrophils, Neutrophil extracellular trap, Extracellular histone-H4/H2A, Microvascular thrombosis, Microglial activation, Neurological recovery

## Abstract

Bacterial pneumonia aggravates ischemic stroke via mechanisms that still remain to be determined. In ischemic stroke patients, we show that stroke-associated pneumonia is associated with poor clinical outcome and long-term neutrophil deregulation. In mice exposed to transient middle cerebral artery occlusion (MCAO), experimental *S. pneumoniae* pneumonia induced at 3 days after stroke impaired neurological recovery and increased blood–brain barrier breakdown, brain neutrophil infiltrates, cerebral microvascular thrombosis, and progressive brain atrophy. The antibiotic amoxicillin only partially ameliorated pneumonia-associated neurological deficits and neutrophil infiltrates. Proteome analysis revealed that pneumonia induced a degranulation, platelet activation, and NETosis signature in peripheral blood neutrophils of MCAO mice, which was partly reversed by amoxicillin treatment. Subsequent studies showed that neutrophils were critical mediators of pneumonia-associated blood–brain barrier breakdown and microvascular thrombosis. Notably, administration of an antibody neutralizing extracellular histone proteins during pneumonia reduced ischemic injury, restored neurological recovery and prevented long-term brain atrophy in stroke-associated pneumonia mice. These protective effects were not observed after degradation of neutrophil extracellular traps by DNase-I, blockade of neutrophil extracellular trap formation by LDC7559 or myeloperoxidase inhibition by AZD4831, when these treatments were administered on occasion of pneumonia. This study identifies extracellular histones as key drivers of inflammatory brain injury and establishes histone neutralization as a strategy that protects against the deleterious effects of pneumonia after stroke.

**Supplementary Information:**

The online version contains supplementary material available at https://doi.org/10.1007/s00401-026-03097-0.

## Introduction

Despite recent progress in recanalizing therapies, ischemic stroke remains the leading cause of long-term disability and second leading cause of death worldwide [43]. In view of this disease burden, there is a major need of treatments that mitigate the long-term consequences of ischemic stroke and enhance recovery in the post-acute stroke phase [16, 20, 28]. Neurorestorative treatments so far failed in randomized controlled clinical trials [7, 12, 17]. Ischemic stroke can trigger various complications, including infections and thrombosis [43, 48]. Due to post-ischemic immunosuppression, ~ 30% of stroke patients suffer infections in acute hospital settings [48]. Pneumonia incidence alone is ~ 8.5–14.3%, and pneumonia incidence of stroke patients on intensive care units is 28% [48]. Pneumonia predisposes to poor stroke outcome: Via the development of sepsis, pneumonia is a leading cause of stroke-associated deaths [8, 14].

Several studies have explored prophylactic antibiotics to prevent stroke-associated pneumonia [22, 49]. In these studies, prophylactic antibiotics consistently did not yield beneficial effects on stroke outcome [22, 49]. Yet, physician-diagnosed pneumonia was independently associated with unfavorable 3-month outcome in one large randomized study [49], while pneumonia diagnosed based on a predefined algorithm was not associated with unfavorable outcome in another one [22]. Some studies have administered β-blockers or interferon-γ to alleviate stroke-induced immunosuppression [5, 19]. Both treatments again did not prevent stroke-associated pneumonia nor enhance stroke outcome [5, 19]. The question whether pneumonia increases ischemic brain damage remains open and underlying mechanisms are still to be explored.

Polymorphonuclear neutrophils are the most abundant leukocytes in humans, which accumulate in the brain parenchyma within hours after ischemic stroke [31, 35]. We previously showed that neutrophils exaggerate ischemic injury and neurological outcome in mice exposed to transient middle cerebral artery occlusion (MCAO) [31]. Recent studies have found that activated neutrophils can release neutrophil extracellular traps (NETs), web-like structures composed of DNA, to which proteins such as myeloperoxidase (MPO) or histones are bound [6]. NETs contribute to host defense by capturing bacterial pathogens [34]. NETs have recently been shown to mediate post-stroke immunosuppression [44]. The role of neutrophils and their secreted contents for neurological outcome in stroke-associated pneumonia was unknown.

We herein asked whether and how stroke-associated pneumonia influences neurological recovery and ischemic injury. Thus, we prospectively examined consequences of stroke-associated pneumonia for neurological recovery in ischemic stroke patients within the “Neutrophils: Origin, Fate and Function” Stroke (NOFF-S) cohort and evaluated the effects of bacterial pneumonia induced by intratracheal *S. pneumoniae* instillation on neurological recovery and ischemic injury in middle cerebral artery occlusion (MCAO) mice. *S. pneumoniae* is the principal cause of bacterial pneumonia in developed and developing countries that is responsible for more infection-related human deaths than any other bacterium [21]. *S. pneumoniae* is one of the most common pathogens in stroke-associated pneumonia. It was therefore the pathogen of choice for us for our experimental studies. After showing that bacterial pneumonia exacerbates neurological outcome and ischemic injury in MCAO mice similar to neurological outcome in stroke patients, we identified underlying neutrophil-driven mechanisms and explored therapeutic strategies to prevent the deleterious effects of pneumonia.

## Materials and methods

### Stroke-associated pneumonia in acute ischemic stroke patients

In the Neutrophils: Origin, Fate & Function Stroke (NOFF-S) study recruited via the German Research Foundation (DFG) Collaborative Research Center (CRC) TRR332 “Neutrophils: origin, fate, and function” (https://www.neutrophils.de, subproject C6) (DRKS00030825), patients with first-ever acute ischemic stroke ≥ 18 years, who were admitted to the Stroke Unit of the University Hospital Essen ≤ 48 h after symptom onset have prospectively been recruited since October 2022. Protocols were approved by the institutional review board of the University Hospital Essen (2110271-BO). All participants provided informed consent. The current data analysis of NOFF-S includes patients included until August 21, 2025. Within 24–< 72 h after admission (examination T1), extensive clinical examinations and structured interviews were performed by a certified study clinician to determine stroke outcome and history of vascular risk factors and diseases. Blood samples were taken for clinical laboratory tests and flow cytometry. Examinations were repeated 72–144 h after admission (T2) and after 3 months (T3). Besides NIHSS and mRS, stroke outcomes included the Barthel Index for the assessment of daily life activities and the Montreal Cognitive Assessment (MoCA). In case patients could not attend the T3 appointment at the University Hospital Essen due to rehabilitation or reduced health condition, telephone-based interviews were conducted to determine mRS. Death certificates were collected to confirm a mRS score of 6.

### Legal issues for animal experimentation, randomization, and blinding

All experiments were performed with local government approval (Landesamt für Verbraucherschutz und Ernährung, Recklinghausen) in accordance to EU guidelines for the care and use of laboratory animals (Directive 2010/63/EU) and are reported in accordance to Animal Research: Reporting of In Vivo Experiments (ARRIVE) guidelines. Experiments were strictly randomized. The experimenters (D.Y., A.R.L.) performing the animal experiments, functional neurological studies, and histochemical studies were fully blinded at all stages of the study by other researchers (S.T., C.W.) preparing injectable solutions. These solutions with dummy names (A and B) were unblinded after termination of the study.

### Animal housing

Mice were housed in a reversed 12-h light/12-h dark cycle, allowing them unrestricted access to food and water. Animals were grouped in cages with 5 animals per cage. Animal surgeries and neurological tests were always performed in the morning hours throughout the study.

### Sample size calculation

Sample size planning was conducted with G*Power version 3.1.9.7 software. Sample size calculations determined that 8 animals per group were required for the main readouts (neurological score, infarct volume, brain edema) to detect group differences with an effect size of 1.3, an α error of 0.05 and a β error of 0.2. For the long-term behavioral studies (tight rope, Rotarod, open field and novel object recognition tests), 14 animals per group were needed to detect group differences with an effect size of 1.0, an α error of 0.05 and a β error of 0.2.

### Intraluminal MCAO model

Focal cerebral ischemia was induced as described before [31, 41]. Male 10–12 week-old C57BL6/j mice (Envigo, Darmstadt, Germany) were anesthetized with 1.5% isoflurane (30% O_2_, remainder N_2_O), while 150 µL of buprenorphine (0.1 mg/kg; Reckitt Benckiser, Slough, U.K.) was injected subcutaneously for analgesia. Rectal temperature was measured and maintained at ~ 37 °C using a feedback-controlled heating system (Fluovac; Harvard apparatus, Holliston, MA, U.S.A.). In all studies, a flexible laser Doppler flow (LDF) probe was attached on the animals’ skulls above the core of the middle cerebral artery territory for regional cerebral blood flow recording (Perimed, Stockholm, Sweden). After a midline neck incision, the left common carotid artery (CCA), internal carotid artery (ICA), and external carotid artery (ECA) were exposed. The CCA and ECA were ligated by sutures, the ICA was temporarily clipped. A silicon-coated 7.0 nylon monofilament (Doccol, Sharon, MA, U.S.A.) was introduced into the CCA through a small incision and advanced up to the bifurcation of the middle cerebral artery. After 30 min of MCAO, the filament was withdrawn to initiate reperfusion. LDF recordings were continued over 15 min. The 30 min MCAO model was the main model, which was used in all interventional experiments within this study. To evaluate a more severe stroke model, we also prepared a set of mice, in which 60 min of MCAO was induced by monofilament insertion through the ECA. The CCA was temporarily clipped in the latter experiments, allowing reopening of CCA blood flow after reperfusion. In all studies, LDF recordings during MCAO and after reperfusion did not differ between groups. The LDF probe was finally removed and all wounds were carefully sutured. During the first 3 days post stroke, animals received daily i.p. injections of carprofen (4 mg/kg; Bayer Vital, Leverkusen, Germany) as anti-inflammatory drug.

### *S. pneumoniae* pneumonia

For bacterial lung infection, a pneumococcal serotype 1 strain (*Streptococcus pneumoniae* SV1, ATCC 33400) was used. Bacteria were grown overnight on Columbia blood agar plates (PB5039A; Thermo Fisher Scientific, Waltham, MA, U.S.A.) at 37 °C. Single colonies were resuspended, cultured in 10 mL brain–heart infusion broth (TV5090E; Thermo Fisher Scientific) to mid-logarithmic phase (OD600 = 0.045–0.055; NanoDrop 1000 spectrophotometer; Thermo Fisher Scientific), and 800 µL of culture supplemented with 200 µL of 86% glycerol was frozen at − 80 °C. For infection, bacteria were again grown to mid-logarithmic phase, centrifuged at 1500*g* at 4 °C for 10 min, and resuspended in 550 µL 0.01 M PBS. 50 µL suspension per animal corresponding to 1 × 10^8^ CFUs *S. pneumoniae* were used for infection [53].

Mice were anesthetized by intraperitoneal injection of ketamine/xylazine (80 mg/kg and 10 mg/kg b.w., respectively). For intratracheal instillation, the mouse was placed on a 60° angled self-made stand and secured by a Velcro stip. The teeth of the mouse were suspended on a thin rubber band and the tongue was pulled out. A 22 gauge cannula with a blunted end was inserted into the mouse trachea with the help of a small-animal laryngoscope (73-4867; Harvard Apparatus, Holliston, MA, U.S.A.). The needle inside was removed and 50 µL of *S. pneumoniae* suspension was pipetted into the cannula. A MiniVent 845 ventilator (73-4867; Harvard Apparatus) was connected to the cannula to evenly spread the suspension into the lungs [53]. In line with earlier studies [53], reproducible neutrophil and macrophage infiltrates were noted in the animals’ lungs on occasion of the animal sacrifice 48 h post-pneumonia in MCAO pneumonia mice.

### Drug administration

As antibiotic, amoxicillin (15 mg/kg; Duphamox LA; Zoetis, Parsipanny, NJ, U.S.A.) dissolved in vehicle (Lipovenös MCT 20%; Fresenius Kabi, Bad Homburg, Germany) or an equivalent volume of vehicle was administered s.c. 3 h post-pneumonia. This injection was repeated at 8 h-intervals until 3 days post pneumonia (dpp), as previously reported [1]. Thus, a total of 9 injections were applied.

To explore the role of neutrophils in the pathological sequelae associated with stroke-associated pneumonia, neutrophils were depleted by i.p. injection of 200 µg of anti-Ly6G antibody (clone 1A8; BioXCell, West Lebanon, NH, U.S.A.) 24 h before pneumonia and 24 h after pneumonia. This dose almost completely clears all neutrophils from the blood, as we previously showed [31]. An equivalent amount of isotype control IgG (clone 2A3; BioXCell) was administered as control treatment.

To degrade circulating NET DNA, deoxyribonuclease-I (DNase-I; 11284932001; Roche, Basle, Switzerland) was applied at a dose of 10 µg intravenously (i.v.) and 50 µg intraperitoneally (i.p.) 30 min before pneumonia. The i.p. dose (50 µg DNase-I) was repeated every 12 h until animal sacrifice, according to previously published protocols [45]. On each occasion, an equivalent amount of vehicle was injected as control treatment.

For NET formation blockade, the gasdermin-D inhibitor LDC7559 (HY-111674; MedChemExpress, Monmouth Junction, NJ, U.S.A.) dissolved in corn oil was injected i.p. at a dose of 10 mg/kg b.w. 30 min before pneumonia in animals sacrificed at 2 dpp, as previously reported [44]. In animals sacrificed at 56 dpp, LDC7559 (10 mg/kg b.w.) was administered i.p. 30 min before pneumonia and at 1, 2, 4, and 7 dpp (animal set A) or immediately after MCAO, 24 h post-MCAO, 30 min before pneumonia and at 1, 2, 4, and 7 dpp (animal set B). For the control group, an equivalent volume of vehicle (corn oil) was applied on each occasion.

To inhibit MPO, the pharmacological inhibitor AZD4831 (HY-145581; MedChemExpress) was administered by oral gavage at a dose of 10 µmol/kg, starting 2 h before pneumonia induction and subsequently once every 24 h until animal sacrifice, as previously published [18]. AZD4831 was dissolved in normal saline containing 1% DMSO, 44% PEG-300, and 5% Tween-80. Control mice received an equal amount of vehicle solution.

To neutralize histone proteins, mice were treated i.p. with 20 µg anti-histone-H4/H2A antibody (clone BWA3; Creative Biolabs, Shirley, NY, USA) [4]. For animals sacrificed at 2 dpp, injections were administered 30 min before pneumonia induction and 1 day later. For animals sacrificed at 56 dpp, injections were performed 30 min before pneumonia induction and at 1, 2, and 3 dpp. Control mice received 20 µg of isotype control IgG (clone MOPC-21; BioXCell, West Lebanon, NH, USA) i.p. at the same time points.

### Neurological score

The modified Clark score is a comprehensive score that examines both general and focal neurological deficits, for which a composite score is formed [41]. This score was studied daily until 7 dpp, subsequently evaluated at 9 dpp, 11 dpp, and 14 dpp, followed by weekly assessments until 56 dpp.

### Tight rope test

The tight rope test consists of a 60-cm-long rope connected to two platforms. Animals were placed on the middle of the rope and the time until reaching one of the platforms or dropping off the rope was recorded [41]. The maximum testing time was 60 s. Recorded times were translated into scores. Animals were trained three times per day for three days before MCAO. After a baseline evaluation before MCAO, animals were tested before pneumonia induction at 0 dpp, and then weekly until 56 dpp.

### Rotarod test

The rotarod is a motor-coordination test that is assessed on a rotating drum [41]. Mice were positioned on the drum, which then began to accelerate from 4 to 40 rpm. The time when the animal dropped off the drum was recorded with a maximum of 300 s for each testing session. The animals were trained 3 times a day for 3 days before the experiments. After a baseline assessment, the mice were evaluated from 0 dpp prior to the induction of pneumonia, followed by weekly assessments up to 56 dpp.

### Open field test

The open field test consists of a square arena (40 × 40 × 30 cm) subdivided into a center, four borders, and four corners, in which animals were placed near the wall and observed for 10 min for evaluating spontaneous motor activity [41]. The total distance covered was tracked using ANY-maze software (Stoelting, Dublin, Ireland). The open field test was performed at 28 dpp and 56 dpp.

### Novel object recognition test

The novel object recognition test uses the same arena as the open field test for assessing recognition memory for presented objects. In a training phase, two identical objects were placed 10 cm away from the walls in opposite corners of the arena. Mice were allowed to explore these objects for 10 min. One hour later, one familiar object was replaced by a novel one of different shape and appearance. Mice were again allowed to explore the chamber for 5 min, while they were tracked using ANY-maze software. Exploration behavior was defined as sniffing, licking, or touching the object with the forepaws. The novel object discrimination index = (novel object exploration time − familiar object exploration time)/(novel object exploration time + familiar object exploration time) was determined.

### Flow cytometry of leukocytes

In human stroke patients, whole blood samples were obtained at the three time-points specified above (T1, T2, and T3; that is, 1–3 days, 4–6 days and 3 months post-stroke). Blood samples were immediately stored at 4 °C and processed within 2 h of collection. For erythrocyte lysis, blood samples were incubated with BD Pharm Lyse buffer at a ratio of 10 mL buffer per 1 mL blood, gently mixed, and incubated for 15 min at room temperature protected from light. Samples were subsequently centrifuged at 900×*g* for 5 min at 4 °C. Supernatants were discarded, and cell pellets were resuspended in PBS containing 1% fetal calf serum (FCS). After an additional washing step, the pellets were resuspended in 1 mL PBS containing 1% FCS. For staining, 15 µL single-cell suspensions were stained for 20 min at 4 °C with antibody cocktails described in Suppl. Table 1. After staining, samples were adjusted to a final volume of 100 µL with PBS containing 1% FCS. Samples were acquired using a CytoFLEX S flow cytometer with CytExpert software version 2.3 (Beckman Coulter, Brea, CA, U.S.A.). Data were analyzed using Kaluza Analysis software version 2.1 (Beckman Coulter).

In MCAO mice, blood samples and ischemic brain hemispheres were collected from the same animals at 48 h post-pneumonia. Single-cell suspensions of blood samples were obtained after erythrocyte lysis and two washing steps. Brain tissue samples were smashed on a 70 μm cell strainer in culture dish, centrifuged with 37% Percoll solution, and after washing single-cell suspensions were obtained. These single-cell suspensions were stained for 30 min at 4 °C with antibody cocktails described in Suppl. Table 2. Subsequently, cell suspensions were analyzed using a Cytoflex flow cytometer (Beckman Coulter) and FlowJo software V10 (Ashland, OR, U.S.A.) using the gating strategy outlined in Suppl. Figure 1.

### Animal sacrifice and measurement of infarct volume, brain edema, atrophy, and hemorrhages

Animals were excluded from the study when they met one of the following criteria: (1) prolonged surgery duration > 20 min; (2) drop of blood flow < 75% after monofilament insertion; (3) lack of reperfusion after monofilament withdrawal; (4) severe weight loss (> 20% of baseline), respiratory abnormalities (central apneas) or death within 3 days after MCAO or MCAO/pneumonia. Animals excluded at various stages of the experiments are summarized in Suppl. Table 3.

At indicated time points, bronchoalveolar lavage fluid (BALF), whole blood and EDTA plasma samples were harvested. Mice were transcardially perfused with ice-cold 0.01 M PBS followed by 4% paraformaldehyde in 0.01 M PBS during 3% isoflurane anesthesia (30% O_2_, remainder N_2_O). After overnight post-fixation in 4% paraformaldehyde in 0.01 M PBS at 4 °C, brains were dehydrated using 15% and 30% sucrose, frozen, and stored at − 80 °C. Then, 20 µm thick coronal sections were collected at 1 mm intervals across the forebrain for cresyl violet staining. Sections were scanned and analyzed by ImageJ software (National Institute of Health, Bethesda, MD, U.S.A.). In animals sacrificed at 2 dpp, infarct volume was determined by subtracting the volume of healthy brain tissue of the ischemic hemisphere from that of the contralateral hemisphere [31]. Brain edema was assessed as increase in volume of the ipsilateral brain hemisphere compared to that of the contralateral hemisphere divided by the volume of the contralateral hemisphere [31]. In animals sacrificed at 56 dpp, brain tissue atrophy was evaluated by the volume reduction of the ipsilateral hemisphere relative to the contralateral hemisphere divided by the volume of the contralateral hemisphere [41].

To analyze brain hemorrhages, 20-µm-thick coronal sections harvested at various levels of the infarct (+ 1.0, ±0.0, − 1.0, and − 2.0 mm rostral to bregma) were incubated in 0.05% diaminobenzidine (DAB) solution (D5905; Sigma-Aldrich). This substrate, oxidized by erythrocyte peroxidases, yields a dark brown staining. The area and incidence of brain hemorrhages were assessed at all levels [51]. The area values determined were extrapolated to volume values by multiplying areas with section thickness values and integrating the values measured across the brain, considering that one of fifty consecutive brain sections was evaluated.

### Brain immunohistochemistry

20-µm-thick coronal sections from the bregma level were rinsed with 0.01 M PBS and permeabilized with 0.3% Triton X-100 in 0.01 M PBS. Subsequently, the sections were incubated in 0.01 M PBS containing 10% normal donkey serum (D9663; Sigma-Aldrich) for 1 h at room temperature. Following that, sections were incubated overnight at 4 °C with primary antibodies. The primary antibodies used were Alexa Fluor-594-conjugated polyclonal donkey anti-IgG (A-21203; Thermo Fisher Scientific), polyclonal goat anti-intercellular adhesion molecule (ICAM-1) (AF796; R&D Systems, Minneapolis, MN, U.S.A.), monoclonal rat anti-CD45 (05-1416; Merck Millipore, Darmstadt, Germany), monoclonal rat anti-Ly6G (lymphocyte antigen 6 locus G; 1A8; 127602; Biolegend, San Diego, CA, U.S.A.), polyclonal rabbit anti-collagen-IV (AB756P; Merck-Millipore), polyclonal goat anti-CD31/PECAM-1 (AF3628; R&D Systems, Minneapolis, MN, U.S.A.), monoclonal rat anti-glycoprotein-Ibα (GP-Ibα) (M043-0; Emfret Analytics, Eibelstadt, Germany), polyclonal rabbit anti-histone-H4 (ab7311; Abcam, Cambridge, U.K.), and polyclonal rabbit anti-ionized calcium binding adaptor protein (Iba)-1 (019–19741; Wako Chemicals, Neuss, Germany) antibodies. Samples were rinsed and labeled with appropriate secondary Alexa Fluor-594 or Alexa Fluor-488 antibodies for 1 h at room temperature. Nuclei were counterstained with Hoechst-33342 (Thermo Fisher Scientific). Sections were coverslipped and scanned using an inverted microscope equipped with apotome optical sectioning (Axio Observer.Z1; Carl Zeiss, Oberkochen, Germany). Extravasated IgG and ICAM-1 were evaluated by integrated signal intensities. For CD45^+^ leukocytes, Ly6G^+^ neutrophils, and Iba1^+^ microglia/macrophages, three 300 μm × 300 μm ROIs were chosen in the ischemic striatum to analyze cell density. For Ly6G^+^ neutrophils, we separately evaluated neutrophils inside or in physical contact with CD31^+^ microvessels and neutrophils at distance to CD31^+^ microvessels. Microvascular thrombosis was assessed by determining the number of collagen-IV^+^ microvessels containing GP-Ibα^+^ clots per mm^2^ in brain sections. Histone-H4 protein deposits were evaluated by analyzing aggregates not associated with Hoechst-33342^+^ cell nuclei. Iba1^+^ TUNEL^+^ microglia/macrophages and Iba1^+^ microglia/macrophages revealing extranuclear histone-H4 deposits were also determined.

### Microglia/macrophage morphology analysis

A Leica SP8 confocal microscope (objective HC PL APO CS2 63 ×/1.30; Leica Microsystems, Wetzlar, Germany) was used to assess the three-dimensional morphology of Iba1^+^ microglia/macrophages in the three representative ROIs. Z-stacks with dimensions of 184.7 × 184.7 × 15 µm were acquired at 0.5 µm intervals. To analyze the 3D cell morphology, our in-house script 3DMorph, based on MATLAB (The MathWorks Inc., Natick, MA, U.S.A.), was utilized. In summary, automated threshold setting and segmentation were employed for the detection of cell objects. Following skeletonization, microglial ramification index and cell territory were obtained.

### Bacterial load in BALF, plasma and brain samples

In BALF, plasma, and ischemic brain tissue samples obtained at 48 h post-pneumonia, bacterial load was measured by serial dilution and CFU counting on Columbia agar with 5% sheep blood (63784; Bio-Rad, Hercules, CA, U.S.A.) after overnight incubation at 37 °C.

### Lung inflammatory responses

Tissue sections were prepared from lungs collected at 48 h post-pneumonia as previously described [37]. After the removal of the Lung, lung was inflated with Histofix (Carl Roth, Karlsruhe, Germany) and then transferred to the fixative. After embedding in paraffin, lung sections with a thickness of 2.5 µm were cut on a microtome. Sections were stained with hematoxylin/eosin (HE) to assess the degree of inflammation, or with periodic acid Schiff (PAS)/HE to determine mucus-producing goblet cells covering the basal membrane, which contribute to infection defense. The degree of lung inflammation was assessed by observers unaware of experimental groups, who scored five randomly selected areas on a scale from 0 (no visible infiltrates around airway vessels and parenchyma) to 4 (several layers of thick cellular infiltrates on nearly all visible vessels and airways). A more detailed score description has been reported [37]. Mucus-producing cells were quantified per 100 µm of basal membrane on three different representative airways on PAS/HE-stained slides.

### Extracellular histone-H4 and citrullinated histone-H3-associated DNA measurement in plasma

Extracellular histone-H4 was quantified in plasma samples collected at 48 h post-pneumonia using a commercially available ELISA kit (EKU08558-96T; Biomatik, Kitchener, ON, Canada). Absorbance was measured at 450 nm, and histone-H4 concentrations were calculated from the standard curve according to the manufacturer’s instructions.

NETs were quantified in plasma samples using a previously described capture ELISA measuring citrullinated histone-H3 (Cit-H3) associated with DNA [44]. Anti-Cit-H3 antibody (5 µg/mL; ab5103; Abcam) was coated overnight in 50 µL volume at 4 °C onto 96-well plates followed by 5% bovine serum albumin (BSA) blocking for 2 h on a shaker at 300 rpm. Coated wells were washed three times with 300 µL washing buffer followed by addition of 50 µL plasma and 80 µL incubation buffer (including peroxidase-labeled anti-DNA antibody) for 2 h at 300 rpm (Cell Death ELISAPLUS; Cat. 11774425001; Roche). Then, the wells were washed three times with 300 µL incubation buffer. Following the addition of 100 µL peroxidase substrate to the wells, samples were incubated in the dark for 30 min at room temperature on a shaker at 300 rpm. Afterward, 100 µL ABTS peroxidase stop solution was added to the wells. Absorbance was measured at 405 nm and absorbance at 490 nm was subtracted. Absorbance values were presented as a relative increase to control.

### Plasma IgA and IgG quantification

IgA and IgG were evaluated in plasma samples obtained at 48 h post-pneumonia using uncoated ELISA kits (IgA: 88-50450, IgG: 88-50400; Thermo Fisher Scientific) according to the manufacturer’s instructions.

### Plasma MPO activity measurement

MPO was determined in plasma samples collected at 24 h post-pneumonia using a colorimetric activity assay (EEA016; Thermo Fisher Scientific) according to the manufacturer’s instructions.

### Isolation of blood neutrophils for proteome analysis

Blood samples were subjected to erythrocyte lysis using a lysis solution (NH_4_Cl 150 mM, KHCO_3_ 10 mM, EDTA 0.1 mM, pH 7.3, 20 °C) with 1 mL used for each sample. The reaction was stopped with 11 mL of ice-cold (4 °C) DMEM after 2 min. Then, the samples were centrifuged at 460*g* at 4 °C for 5 min and the supernatant was removed. Samples were washed with ice-cold (4 °C) 0.1 M PBS thereafter. Following resuspension of the pellet and incubation with Fc-blocking antibodies (CD16/CD32; clone 2.4G2; BD Biosciences, Heidelberg, Germany) at 4 °C for 15 min, samples were incubated with anti-Ly6G antibody (clone 1A8; BD Biosciences), anti-CD11b antibody (clone M1/70; Affymetrix, Santa Clara, CA, U.S.A.), and DAPI (BioLegend). After washing and resuspension, CD11b^+^ Ly6G^+^ live neutrophils were sorted using a FACS Aria cell sorter (BD Biosciences), and the purity of cells was assessed (≥ 95%). The cells were then stored in protein lysis buffer and frozen in − 80 °C until future use.

### Sample preparation for proteomics

The sorted neutrophils (typically 40,000–50,000) were extensively washed with PBS and resuspended in lysis buffer (50 mM Tris–HCl (pH 7.8), 150 mM NaCl, and 1% SDS) supplemented with complete mini-EDTA-free protease inhibitor (Roche, Penzberg, Germany). Each sample was incubated with 2.5 U benzonase nuclease (Merck-Millipore) for 7 min at room temperature. The proteins were reduced for 30 min at 37 °C in 10 mM dithiothreitol (DTT) and alkylated in 30 mM iodoacetamide for 30 min at room temperature in the dark. After that, the proteins were precipitated with nine volumes of ethanol for 2 h at − 80 °C and centrifuged for 45 min at 20,000 g. The supernatant was removed, the pellet was dried and dissolved first in 1 µL of 6 M GuHCl and then in 29 µL of 50 mM ammonium bicarbonate buffer, pH 7.8, containing 2 mM CaCl_2_ and 50 ng of trypsin (sequencing grade; Promega, Fitchburg, WI, U.S.A.), before incubation for 15 h at 37 °C. The concentration of peptides was measured on a Nanodrop spectrophotometer at 205 nm. The enzymatic digestion was stopped by acidifying the sample to pH < 2.5 with TFA.

### LC–MS/MS data acquisition

Chromatographic separation of peptides was achieved with a two-buffer system (buffer A: 0.1% FA in H_2_O, buffer B: 0.1% FA in 80% ACN) on a UHPLC system (VanquishTM neo; Thermo Fisher Scientific). Attached to the UHPLC was a peptide trap (100 µm × 20 mm, 100 Å pore size, 5 µm particle size, C18; Nano Viper; Thermo Fisher Scientific) for online desalting and purification, followed by a 25 cm C18 reversed-phase column (75 µm × 250 mm, 130 Å pore size, 1.7 µm particle size; peptide BEH C18; nanoEase; Waters, Eschborn, Germany). Peptides were separated using a 70 min protocol with linearly increasing ACN concentration from 2.5 to 37.5% ACN over 60 min.

MS/MS measurements were performed on a quadrupole-orbitrap hybrid mass spectrometer (Exploris 480; Thermo Fisher Scientific). Eluting peptides were ionized using a nano-electrospray ionization source (nano-ESI) with a spray voltage of 1,800 and analyzed in data dependent acquisition (DDA) mode. For each MS1 scan, ions were accumulated for a maximum of 25 ms or until a charge density of 3 × 10^6^ ions (AGC Target) was reached. Fourier-transformation based mass analysis of the data from the orbitrap mass analyzer was performed covering a mass range of m/z 350–1400 with a resolution of 60,000 at m/z = 200. Peptides being responsible for the 20 highest signal intensities per precursor scan with a minimum intensity of 8 × 10^3^ and charge state from + 2 to + 6 were isolated within a m/z 2 isolation window and fragmented with a normalized collision energy of 30% using higher energy collisional dissociation (HCD). MS2 scanning was performed, covering a mass range starting at m/z 120 and accumulated for 50 ms or to an AGC target of 5 × 10^4^ at a resolution of 15,000 at m/z = 200. Already fragmented peptides were excluded for 30 s.

### Raw data processing

LC–MS/MS were searched with the Sequest algorithm integrated in the Proteome Discoverer software (v 3.0.0.757; Thermo Fisher Scientific) against a reviewed Mus musculus database obtained in December 2022. Carbamidomethylation was set as fixed modification for cysteine residues and the oxidation of methionine as well as methionine-loss and acetylation of the protein N-terminus were allowed as variable modifications. A maximum number of 2 missing tryptic cleavages was set. Peptides between 6 and 144 amino acids where considered. A strict cutoff (FDR<0.01) was set for peptide and protein identification. Quantification was performed using the Minora Algorithm, implemented in Proteome Discoverer. Results were filtered for high confident peptides, with enhanced peptide and protein annotations. Protein abundances were exported for subsequent statistical analysis and normalized.

### Data analysis, statistics, and visualization

Statistical analyses were conducted using GraphPad Prism version 9.5.1 for Windows (GraphPad Software, San Diego, CA, U.S.A.). LDF recordings, neurological deficits, and behavioral tests were analyzed by repeated measures ANOVA, with subsequent LSD posthoc test. These longitudinal data were presented as mean ± standard deviation (SD). For histochemical and proteomics data that were normally distributed, two-tailed independent samples t-tests or one-way analysis of variance (ANOVA) followed by LSD posthoc tests were utilized, as appropriate. For data that did not follow a normal distribution, the Kruskal–Wallis test followed by Dunn’s tests was used. These cross-sectional data were presented in box plots containing median and mean values ± interquartile ranges (IQR) with minimum and maximum data as whiskers. Hemorrhage incidence data were assessed by Fisher’s exact test and reported as percent rates. P values < 0.05 were considered to indicate statistical significance.

LC–MS/MS data were filtered by removing samples with low abundance and proteins that were not found in at least 3 samples of one group and subsequently normalized using loess normalization (R-limma) followed by imputation with a uniform random 5th–10th percentile approach for missing values. Statistical significance was tested using Student t-test (scipy). Effect sizes were computed with signal-to-noise ratio (SNR) and log_2_ fold change (FC) where the log_2_ FC is determined as ${log}_{2}\mathrm{F}\mathrm{C}={\mathrm{log}}_{2}\left(\frac{\overline{{\mathrm{x} }_{1}}}{\overline{{\mathrm{x} }_{2}}}\right)$, with $\overline{{\mathrm{x} }_{1}}$ and $\overline{{\mathrm{x} }_{2}}$ as the mean values, and the SNR is calculated using $SNR=\left(\frac{\overline{{x }_{1}}-\overline{{x }_{2}}}{{d}_{1}+{d}_{2}}\right)$ with d_1_ and d_2_ as the respective standard deviation. Significant regulation was considered for the proteins with an absolute log_2_ fold change > 0.263 and p value < 0.05. Heatmaps, volcano plots, and principal component analysis (PCA) were generated using Morpheus (https://software.broadinstitute.org/morpheus), GraphPad Prism version 9.5.1 for Windows, the Python package matplotlib (v.3.8.1) and seaborn (v.0.13.2).

## Results

### Stroke-associated pneumonia is associated with poor clinical outcome and long-term neutrophil deregulation

To characterize the effect of stroke-associated pneumonia in acute ischemic stroke patients, we prospectively examined clinical outcome in the prospective NOFF-S cohort. Patients experiencing stroke-associated pneumonia (26 patients) during their hospital stay had similar age and sex distribution as patients not experiencing pneumonia (326 patients), but more severe strokes at admission defined by the National Institute of Health Stroke Scale (NIHSS) score (Table 1). Stroke outcome defined by the modified Rankin Scale (mRS) score at 90 days was worse in stroke patients with pneumonia than stroke patients without pneumonia (Table 1). Pneumonia predicted poor stroke outcome at 90 days in univariate analyses (relative risk (RR) 3.2 (1.0–9.9), p = 0.044) and multivariable analyses adjusted for age and sex (3.2 (1.0–9.9), p = 0.045). RR only modestly decreased to 2.3 (0.7–7.4) after age, sex, and NIHSS adjustment.

**Table 1:** Baseline characteristics and clinical outcome of ischemic stroke patients with and without stroke-associated pneumonia in the NOFF-S cohort

Variables	Stroke-associated pneumonia (n = 26 patients)	No stroke-associated pneumonia (n = 326 patients)	p
Demographics			
Age	70.0 (61.5;79.5)	70.0 (60.0;78.3)	0.784
Male sex	15 (57.7)	198 (60.7)	0.760
Stroke severity			
NIHSS at admission	12.0 (9.5;15.0)	4.0 (2.0;7.0)	< 0.001
NIHSS T1	11.0 (6.3;14.8)	3.0 (1.0;5.0)	< 0.001
mRS T1	5.0 (4.0;5.0)	2.0 (1.0;3.5)	< 0.001
Barthel Index T1	20.0 (5.0;40.0)	85.0 (60.0;100.0)	< 0.001
MoCA T1	17.0 (11.0;20.0)	22.0 (18.0;25.0)	0.013
NIHSS T2	10.0 (5.0;13.0)	2.0 (1.0;4.0)	< 0.001
mRS T2	4.0 (4.0;5.0)	2.0 (1.0;3.0)	< 0.001
Barthel Index T2	20.0 (10.0;42.5)	95.0 (75.0;100.0)	< 0.001
MoCA T2	17.0 (15.0;20.0)	22.0 (18.0;26.0)	0.001
Stroke subtype			
Large artery atherosclerosis	9 (36.0)	107 (33.4)	
Cardioembolic	12 (48.0)	83 (25.9)	
Small artery occlusion	3 (12.0)	49 (15.3)	
Other causes	0 (0.0)	15 (4.7)	
Undefined	1 (4.0)	66 (20.7)	0.076
Cardiovascular risk factors			
Arterial hypertension	17 (65.4)	247 (76.2)	0.216
Diabetes mellitus	7 (26.9)	92 (28.4)	0.873
Dyslipidemia	13 (50.0)	163 (50.3)	0.976
Atrial fibrillation	13 (50.0)	69 (21.3)	< 0.001
Current smoking	3 (15.0)	75 (25.2)	0.283
BMI	27.1 (24.4;32.7)	26.6 (23.5;30.5)	0.630
Cardiovascular diseases			
History of myocardial infarction	2 (7.7)	27 (8.3)	0.909
Coronary artery disease	5 (19.2)	60 (18.5)	0.721
Peripheral artery disease	4 (8.2)	13 (4.3)	0.928
Interventions			< 0.001
Thrombolysis	3 (11.5)	77 (23.8)	
Thrombectomy	6 (23.1)	14 (4.3)	
Thrombolysis plus thrombectomy	6 (23.1)	27 (8.3)	
None	11 (42.3)	206 (63.6)	
Clinical outcomes			
mRS T3	2.0 (1.0;4.0)	1.0 (0.0;2.0)	0.043
NIHSS T3	1.0 (1.0;1.0)	1.0 (0.0;1.0)	0.144
Barthel Index T3	100.0 (90.0;100.0)	100.0 (100.0;100.0)	0.154
MoCA T3	24.0 (23.0;24.0)	25.0 (22.0;27.0)	0.368

Unless otherwise indicated, data are medians (Q1;Q3) or numbers (%). P-values were assessed by Mann–Whitney U tests for continuous data, categorical data by Chi-square or Fisher’s exact tests. T1: 24–72 h after admission, T2: 72–144 h after admission, T3: after 3 months

Flow cytometry analyses of blood samples revealed that stroke patients experiencing pneumonia exhibited very similar leukocyte and neutrophil numbers as patients not experiencing pneumonia shortly after their hospital admission, i.e., at 1–3 days post-stroke (Table 2). The percentage of activated CD15^high^CD62L^int^ neutrophils at this early time-point was lower in stroke patients developing pneumonia than patients not developing pneumonia (Table 2). This pattern shifted within 4–6 days post-stroke (which is the typical time-window of pneumonia manifestation), when the absolute number of leukocytes and neutrophils became nominally, but not statistically significantly higher in patients exhibiting pneumonia than patients not exhibiting pneumonia. The percentage of activated CD15^high^CD62L^int^ neutrophils at this time-point no more differed between the two groups, but the percentage of CD15^low^CD62L^high^ and CD15^low^CD62L^int^ neutrophils was lower in patients with pneumonia than patients without pneumonia (Table 2). This pattern persisted in the post-acute phase at 3 months post-stroke. Due to their reduced tissue infiltration, neutrophils with low CD15 expression were shown to predispose to high bacterial load and severe lung damage in patients with pneumonia [30], and in the spleen CD15^low^ neutrophils were proposed to have immunosuppressive properties as B cell helper cells [36]. The long-lasting neutrophil deregulation raised our attention for the role of neutrophils in the recovery of stroke associated with pneumonia.

**Table 2:** Flow cytometry of ischemic stroke patients with and without stroke-associated pneumonia

Variables	Stroke-associated pneumonia (n = 17 patients)	No stroke-associated pneumonia (n = 219 patients)	p
	T1		
Leukocytes/mL	7.41 ± 6.49 × 10⁶	7.73 ± 10.10 × 10⁶	0.905
Neutrophils/mL	6.00 ± 5.42 × 10⁶	5.12 ± 6.45 × 10⁶	0.690
CD15^high^CD62L^high^ neutrophils (%)	63.6 ± 27.2	57.7 ± 22.0	0.565
CD15^high^CD62L^int^ neutrophils (%)	10.0 ± 8.2	20.3 ± 21.9	0.010
CD15^low^CD62L^high^ neutrophils (%)	8.3 ± 16.7	2.3 ± 6.3	0.348
CD15^low^CD62L^int^ neutrophils (%)	18.1 ± 15.3	19.7 ± 20.9	0.784
	T2		
Leukocytes/mL	7.62 ± 6.63 × 10⁶	5.51 ± 7.25 × 10⁶	0.198
Neutrophils/mL	6.00 ± 5.14 × 10⁶	3.63 ± 5.05 × 10⁶	0.076
CD15^high^CD62L^high^ neutrophils (%)	65.2 ± 18.4	57.7 ± 25.5	0.130
CD15^high^CD62L^int^ neutrophils (%)	23.2 ± 21.7	22.8 ± 24.2	0.941
CD15^low^CD62L^high^ neutrophils (%)	1.7 ± 1.6	3.5 ± 10.5	0.022
CD15^low^CD62L^int^ neutrophils (%)	9.9 ± 10.1	16.0 ± 21.6	0.041
	T3		
Leukocytes/mL	8.90 ± 8.88 × 10⁶	4.72 ± 8.01 × 10⁶	0.503
Neutrophils/mL	5.12 ± 4.90 × 10⁶	3.05 ± 5.22 × 10⁶	0.544
CD15^high^CD62L^high^ neutrophils (%)	76.4 ± 20.1	58.1 ± 23.7	0.254
CD15^high^CD62L^int^ neutrophils (%)	8.1 ± 8.5	23.6 ± 23.1	0.073
CD15^low^CD62L^high^ neutrophils (%)	0.3 ± 0.2	3.7 ± 12.3	< 0.001
CD15^low^CD62L^int^ neutrophils (%)	15.2 ± 25.3	14.6 ± 20.6	0.972

Data are mean ± SD numbers. p-values were assessed by two-tailed t-tests with Welch’s corrections. T1: 24–72 h after admission, T2: 72–144 h after admission, T3: after 3 months

### Pneumonia exacerbates neurological deficits, microvascular permeability, and thromboinflammation in ischemic mice

**Fig. 1:**
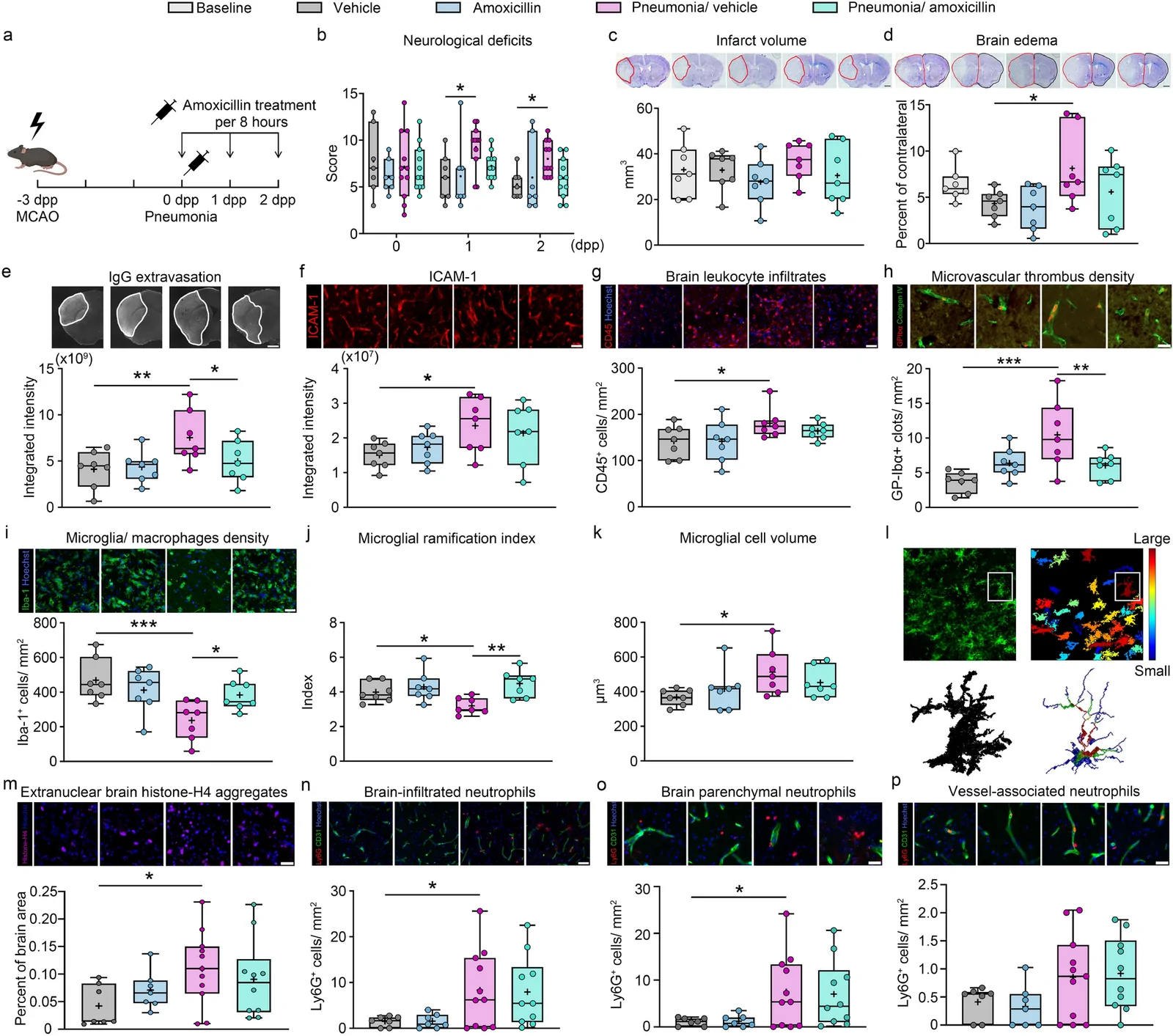
Effects of bacterial pneumonia on post-stroke neurological deficits, blood–brain barrier (BBB) breakdown, microvascular thrombosis, and brain inflammatory responses are incompletely reversed by amoxicillin. **a** Time line of animal experiments, **b** neurological deficits assessed by a comprehensive behavioral score, **c** infarct volume, and **d** brain edema evaluated by cresyl violet staining, **e** IgG extravasation in ischemic brain tissue, **f** ICAM1 expression on ischemic microvessels, **g** brain infiltrated CD45^+^ leukocytes, **h** microvascular thrombus density in ischemic brain tissue, **i–k** density and activation of Iba1^+^ microglia/macrophages in the ischemic striatum (i.e., the core of the middle cerebral artery vascular territory), assessed by cell counting and morphology analysis following cellular segmentation, skeletonization, and reconstruction (a representative cell segmentation and skeleton is shown in **l**), **m** extranuclear histone-H4 deposits in the ischemic striatum (in red; Hoechst-33342^+^ nuclei shown in purple), as well as **n** total brain-infiltrated Ly6G^+^ neutrophils, **o** brain parenchymal Ly6G^+^ neutrophils, and** p** vessel-associated Ly6G^+^ neutrophils (in red; CD31^+^ microvessels shown in green) of mice subjected to 30 min intraluminal MCAO, which were not exposed to pneumonia or exposed to pneumonia induced by intratracheal *S. pneumoniae* instillation (1×10^8^ CFUs) at 3 days post-MCAO, followed by vehicle or amoxicillin (15 mg/kg t.i.d., s.c.) treatment starting 3 h thereafter. Mice were sacrificed at 2 days post-pneumonia (dpp; i.e., 5 days post-MCAO). In **b–d**, mice not exposed to pneumonia sacrificed at 3 days post-MCAO (the ‘baseline’ time point of pneumonia induction) are also shown. Representative cresyl violet and immunohistochemistry images are depicted. *p < 0.05, **p < 0.01, ***p < 0.001 (n = 7–11 mice/group). Scale bars: 1 mm (in **c–e**); 50 μm (in **f**, **g**, **n**); 30 μm (in **h**, **I**, **m**, **o**, **p**). Mouse illustration in **a** created in BioRender.com

To explore the neuropathological correlates associated with stroke-associated pneumonia, we exposed mice to transient 30 min intraluminal MCAO and induced experimental pneumonia 72 h later by intratracheal *S. pneumoniae* instillation (time line in Fig. 1a). Experimental pneumonia decreased rectal temperature for up to 12 h (Suppl. Figure 2a), decreased body weight by ~ 10% (Suppl. Figure 2b), and increased neurological deficits at 1 and 2 days post-pneumonia (dpp) (Fig. 1b). Analysis of colony forming units revealed a reproducible load of *S. pneumoniae* in the BALF of MCAO/pneumonia mice at 2 dpp (Suppl. Figure 2c), but absence of colony-forming units in the blood and brain (Suppl. Figure 2d, e). Histological analysis revealed reproducible inflammatory responses in the lungs, as revealed by HE and PAS staining (Suppl. Figure 2f, g).

Pneumonia did not influence infarct volume at 2 dpp (that is, 5 days post-MCAO) (Fig. 1c), but significantly increased post-ischemic brain edema (Fig. 1d) and IgG extravasation (Fig. 1e), which are markers of blood–brain barrier (BBB) breakdown. Pneumonia also increased intercellular adhesion molecule-1 (ICAM1) abundance on cerebral microvessels (Fig. 1f), increased brain CD45^+^ leukocyte infiltrates (Fig. 1g), increased the density of GP-Ibα^+^ microvascular thrombi (Fig. 1h), reduced the density of Iba1^+^ microglia/macrophages (Fig. 1i), increased microglial/macrophage activation (evidenced by a reduced ramification index; Fig. 1j–l), and increased extranuclear histone-H4 deposits (Fig. 1m), an inflammation driver [32, 40], including microglial extranuclear histone-H4 deposits (Suppl. Figure 2 h), in the previously ischemic brain tissue. IgG extravasation, ICAM1 abundance on cerebral microvessels, brain CD45^+^ leukocyte infiltrates, GP-Ibα^+^ microvascular thrombus density, Iba1^+^ microglia/macrophage density and microglia/macrophage activation in the homologous contralateral brain tissue were not influenced by pneumonia (Suppl. Figure 3a–g).

In order to test the validity of these results in a model of more severe ischemia, we exposed mice to transient 60 min MCAO and again induced *S. pneumoniae* pneumonia 72 h later followed by animal sacrifice at 2 dpp (Suppl. Figure 4a). As in the less severe ischemic stroke model, experimental pneumonia increased neurological deficits at 1 and 2 dpp (Suppl. Figure 4b). Again, pneumonia did not influence infarct volume at 2 dpp (that is, 5 days post-MCAO) (Suppl. Figure 4c), but increased brain edema, brain CD45^+^ leukocyte infiltrates, and GP-Ibα^+^ microvascular thrombus density, reduced Iba1^+^ microglia/macrophage density and increased microglia/macrophage activation (Suppl. Figure 4d–l). Thus, stroke-associated pneumonia induced a robust surrogate of structural brain injury that in both MCAO models was associated with neurological deterioration.

### Antibiotic amoxicillin incompletely reverses the pneumonia-associated neurological deficits and microvascular inflammation

Antibiotic treatment with amoxicillin initiated 3 h post-pneumonia attenuated the rectal temperature decrease associated with pneumonia in 30 min MCAO mice (Suppl. Figure 2a). Body weight was not significantly influenced by amoxicillin (Suppl. Figure 2b). Amoxicillin significantly reduced the load of *S. pneumoniae* in the BALF of MCAO/pneumonia mice (Suppl. Figure 2c), but modestly increased inflammatory responses in the lungs at 2 dpp, as revealed by HE (Suppl. Figure 2f).

In MCAO/pneumonia mice, amoxicillin did not significantly affect neurological deficits (Fig. 1b) or brain infarct volume at 2 dpp (that is, 5 days post-MCAO) (Fig. 1c), but attenuated the pneumonia-associated BBB breakdown (Fig. 1d, e), but not ICAM1 levels on cerebral microvessels (Fig. 1f) or brain CD45^+^ leukocyte infiltrates at 2 dpp (Fig. 1g). Amoxicillin reduced the GP-Ibα^+^ microvascular thrombus density in the previously ischemic brain tissue of MCAO/pneumonia mice (Fig. 1h). Besides, amoxicillin restored the density of Iba1^+^ microglia/macrophages, and reduced their activation, but did not influence extranuclear histone-H4 deposits, assessed by histone-H4/Hoechst-33342 colabeling (Fig. 1i–m; Suppl. Figure 2 h). BBB breakdown, brain inflammatory responses, microvascular thrombosis, and microglial/macrophage density and activation were not influenced by amoxicillin in the homologous contralateral brain tissue (Suppl. Figure 3a–g). Hence, antibiotic treatment incompletely reversed the neurological worsening and histopathological injury associated with pneumonia, in line with randomized controlled trials in stroke patients, in which antibiotics failed to enhance stroke outcome [22, 49].

### Amoxicillin does not reverse the pneumonia-associated neutrophil responses in the ischemic brain

To elucidate mechanisms underlying the pneumonia-associated brain damage, we performed flow cytometry of leukocytes obtained from peripheral blood and ischemic brain tissue at 2 dpp using the sorting strategy provided in Suppl. Figure 1. In these and all subsequent studies, mice were again exposed to 30 min MCAO. Pneumonia did not significantly alter the overall number of CD45^+^ leukocytes in peripheral blood, but significantly increased the number and activation of Ly6G^+^ neutrophils and CD8^+^ T cell activation (Suppl. Figure 5). Amoxicillin reversed the elevated neutrophil number and activation and reduced the CD4^+^ and CD8^+^ T cell number in the blood of MCAO/pneumonia mice (Suppl. Figure 5). Plasma IgA and IgG, which are markers of adaptive immune function [44], were not influenced by amoxicillin (Suppl. Figure 6a, b), as were extracellular histone-H4 protein and Cit-H3-associated DNA in the plasma (Suppl. Figure 6c, d).

**Fig. 2:**
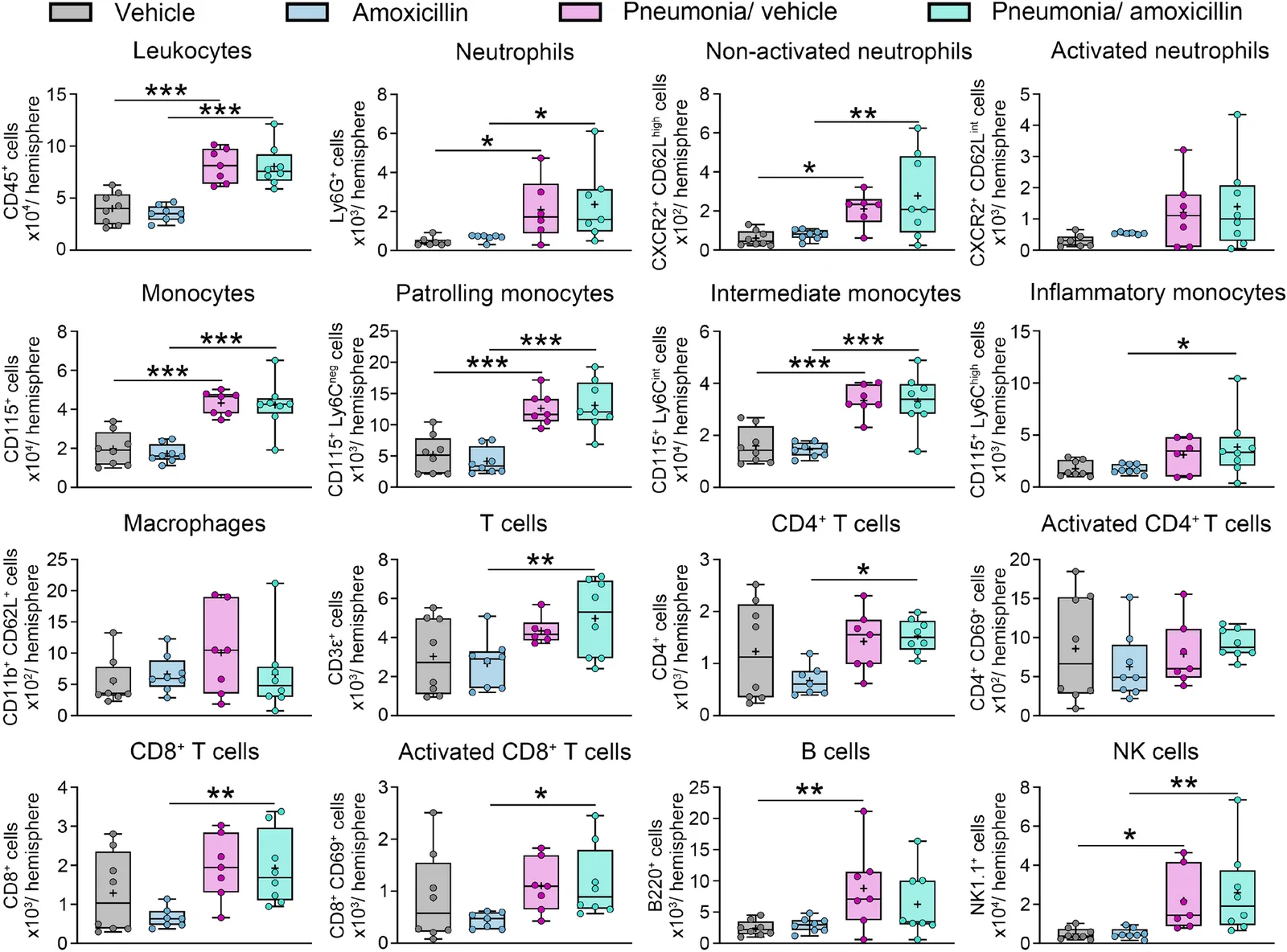
Pneumonia increases the brain infiltration of neutrophils, monocytes, T cells, B cells, and NK cells, which is not reversed by amoxicillin. Number of infiltrated CD45^+^ leukocytes, Ly6G^+^ neutrophils, Ly6G^+^ CXCR2^+^ CD62L^high^ non-activated neutrophils, Ly6G^+^ CXCR2^+^ CD62L^int^ activated neutrophils, CD115^+^ monocytes, CD115^+^ Ly6C^neg^ patrolling monocytes, CD115^+^ Ly6C^int^ intermediate monocytes, CD115^+^ Ly6C^high^ inflammatory monocytes, CD115^−^ CD11b^+^ CD62L^+^ macrophages, CD3ε^+^ T cells, CD4^+^ T cells, CD4^+^ CD69^+^ activated T cells, CD8^+^ T cells, CD8^+^ CD69^+^ activated T cells, B220^+^ B cells, and NK1.1^+^ NK cells in the ischemic brain assessed by flow cytometry in MCAO mice not exposed to pneumonia or MCAO mice exposed to bacterial pneumonia at 3 days post-MCAO, which were treated with vehicle or amoxicillin (15 mg/kg t.i.d., s.c.) starting 3 h later. Animals were sacrificed at 2 dpp. *p < 0.05, **p < 0.01, ***p < 0.001 (n = 7–8 mice/group)

Pneumonia significantly increased the number of infiltrated CD45^+^ leukocytes, including Ly6G^+^ neutrophils, CD115^+^ monocytes (particularly patrolling and intermediate monocytes), CD3ε^+^ T cells, B220^+^ B cells, and NK1.1^+^ NK cells, in the ischemic brain tissue (Fig. 2). Of note, brain-infiltrated leukocyte numbers, including neutrophil numbers, were not influenced by amoxicillin (Fig. 2). To further explore neutrophil responses in the ischemic brain tissue, we evaluated neutrophil distribution by immunohistochemistry by Ly6G doublelabeling with the endothelial marker CD31. These studies confirmed that the total number of brain-infiltrated Ly6G^+^ neutrophils was significantly increased by pneumonia, but not influenced by antibiotic treatment with amoxicillin (Fig. 1n). Pneumonia more strongly increased the number of brain parenchymal neutrophils than vessel-associated neutrophils (Fig. 1o, p). Neither brain parenchymal nor vessel-associated neutrophils were reduced by amoxicillin.

### Pneumonia induces a degranulation, platelet activation, and NETosis signature in blood neutrophils

**Fig. 3:**
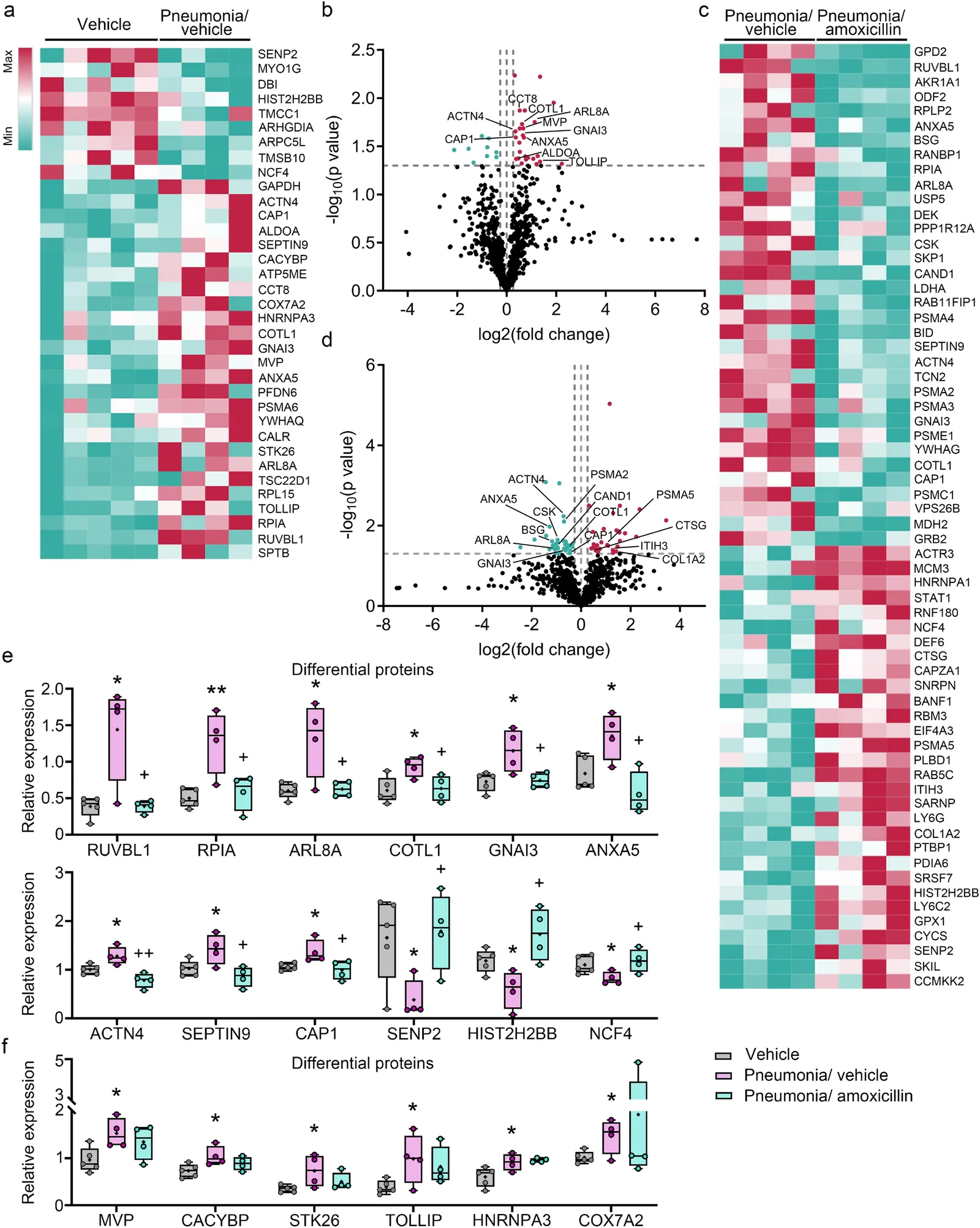
Pneumonia induces a neutrophil degranulation and NETosis signature that is reversed by amoxicillin. **a** Heatmap and **b** volcano plot of differentially expressed proteins in peripheral blood neutrophils of MCAO mice exposed to bacterial pneumonia at 3 days post-MCAO treated with vehicle (s.c.) compared with MCAO mice without pneumonia treated with vehicle which were sacrificed at 2 dpp. **c** Heatmap and **d** volcano plot of differentially expressed proteins of blood neutrophils of MCAO/pneumonia mice treated with amoxicillin (15 mg/kg t.i.d., s.c.) starting 3 h post-pneumonia compared with MCAO/pneumonia mice treated with vehicle which were sacrificed at 2 dpp. **e** Proteins significantly regulated by pneumonia and amoxicillin in opposing ways. Note that besides proteins involved in neutrophil degranulation (ARL8A, COTL1, CAP1) and platelet activation (GNAI3, ANXA5, ACTN4, CAP1), the histone 2B family protein HIST2H2BB and RUVBL1 protein that promotes chromatin decondensation, histone modification, and chromatin remodeling were identified. **f** Proteins significantly regulated by pneumonia, but not influenced by amoxicillin. Among these proteins were CACYBP, a calcyclin-binding protein involved in protein ubiquination and proteasomal degradation, STK26, a Golgi-associated serine-threonine kinase involved in the regulation of apoptosis, and COX7A2, a cytochrome-C oxidase component relevant for mitochondrial electron transport. Particularly notable is the increase of TOLLIP (toll-interacting protein) in response to pneumonia. TOLLIP is a positive regulator of chronic inflammation under conditions of atherosclerosis [25] and exacerbates brain injury in ischemic stroke [27]. Protein levels in **e**, **f** were normalized to sham control. *p < 0.05, **p < 0.01 for MCAO/pneumonia/vehicle compared with MCAO only/vehicle; ^+^p < 0.05, ^++^p < 0.01 for MCAO/pneumonia/amoxicillin compared with MCAO/pneumonia/vehicle (n = 4–5 mice/group)

To further explore the role of neutrophils in stroke-associated pneumonia, we collected blood samples of MCAO mice receiving vehicle, MCAO/pneumonia mice receiving vehicle or MCAO/pneumonia mice receiving amoxicillin at 2 dpp (that is, 5 days post-MCAO), from which neutrophils were isolated for proteome analysis. In total, 1058 proteins were detected in neutrophil samples. In a comparison between the MCAO/pneumonia/vehicle and the MCAO only/vehicle group, 35 proteins with p-values less than 0.05 and absolute log_2_ (fold change) larger than 0.263 were identified as differentially expressed, was shown by a heatmap of their relative expression levels (Fig. 3a). Among these 35 proteins, 26 were upregulated and 9 were downregulated by pneumonia. Among the proteins increased by pneumonia, 7 were related to neutrophil degranulation (CCT8, COTL1, MYP, ARL88, TOLLIP, CAP1, ALDOA) and 5 related to platelet activation (CAP1, ALDOA, ACTN4, GNAI3, ANXA5) (Fig. 3b).

**Fig. 4:**
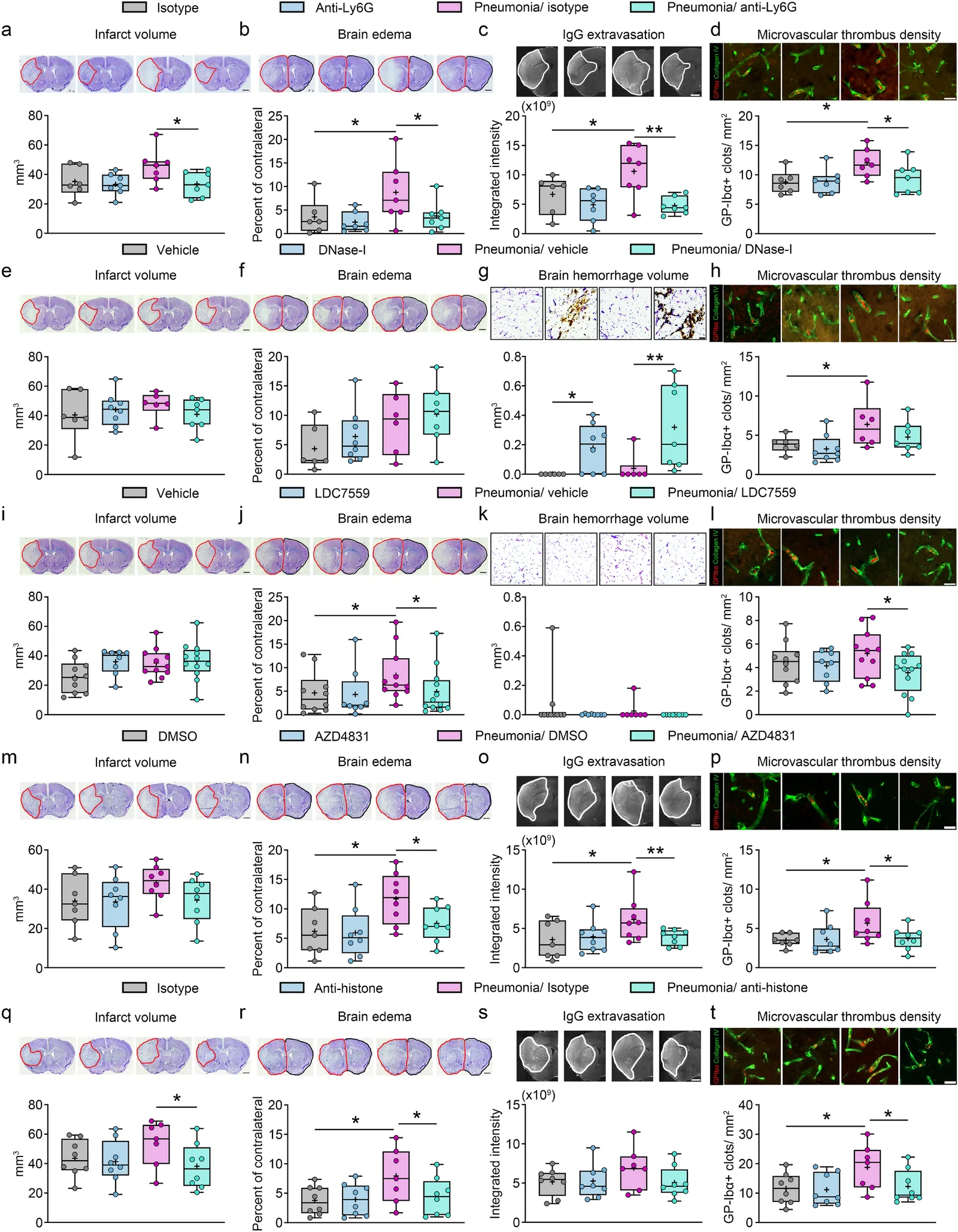
Antibody-mediated neutrophil depletion, NET formation blockade, MPO inhibition, and extracellular histone neutralization reverse the early neuropathological sequelae associated with pneumonia. **a**, **e**, **i**, **m**, **q** Infarct volume, **b**, **f**, **j**, **n**, **r** brain edema, **c**, **o**, **s** IgG extravasation, **d**, **h**, **l**, **p**, **t** GP-Ibα^+^ thrombus density (in red) on collagen-IV^+^ cerebral microvessels (in green), and **g**, **k** brain hemorrhage volume (hemorrhages in brown; cresyl violet counterstaining in blue) in the previously ischemic brain tissue of MCAO mice not exposed to pneumonia or MCAO mice exposed to bacterial pneumonia at 3 days post-MCAO, which were treated with **a–d** isotype antibody or anti-Ly6G antibody (1A8; 200 µg i.p.), which near-completely depletes neutrophils [31], 1 day before and 1 day after pneumonia (i.e., at 2 and 4 days post-MCAO), **e–h** vehicle or DNase-I (10 µg i.v. and 50 µg i.p. 30 min before pneumonia, followed by 50 µg b.i.d.), which degrades NETs [44], **i–l** vehicle or the gasdermin-D inhibitor LDC7559 (10 mg/kg i.p.), which prevents NET formation [44], 30 min before pneumonia and 1 day after pneumonia,** m–p** vehicle or the MPO inhibitor AZD4831 (10 µmol/kg via oral gavage) 2 h before and 1 day after pneumonia, or **q–t** isotype IgG or neutralizing anti-histone-H4/H2A antibody (BWA3; 20 µg i.p.) 30 min before and 1 day after pneumonia, followed by animal sacrifice at 2 dpp (that is, 5 days post-MCAO). Representative cresyl violet and immunohistochemistry images are shown. *p < 0.05, **p < 0.01 (n = 6–12 mice/group). Scale bars: 1 mm (in **a–c**, **e**, **f**, **i**, **j**, **m–o**, **q–s**); 30 µm (in **d**, **h**, **l**, **p**, **t**); 50 μm (in **g**, **k**)

In a comparison between the MCAO/pneumonia/amoxicillin and MCAO/pneumonia/vehicle groups, 64 proteins were differentially expressed with p-values less than 0.05 and absolute log_2_ (fold change) larger than 0.263, with 34 downregulated and 30 upregulated proteins (Fig. 3c). Among the 34 downregulated proteins, 5 were related to neutrophil degranulation (CAND1, PSMA2, COTL1, ARL8A, CAP1), 5 to platelet activation (CAP1, ANXA5, CSK, ACTN4, GNAI3), and one to cell and vascular wall adhesion (BSG) (Fig. 4d). Besides, 2 proteins related to neutrophil degranulation (CTSG, PSMA5) and 2 associated with platelet activation (ITIH3, COL1A2) were increased by amoxicillin (Fig. 3d).

12 proteins were oppositely regulated by pneumonia and amoxicillin (Fig. 3e). Of these, 9 proteins were increased by pneumonia and decreased by amoxicillin. Three of these proteins were related to neutrophil degranulation (ARL8A, COTL1, CAP1) and 4 to platelet activation (GNAI3, ANXA5, ACTN4, CAP1). One histone modification and 1 histone protein were also conversely regulated in the three conditions: RUVBL1, a RUVB-like-1 protein that promotes chromatin decondensation, histone modification, and ATP-dependent chromatin remodeling (UniProt #P60122), and HIST2H2BB, a histone 2B family protein (UniProt #P62806) (Fig. 3e). We interpreted the RUVBL1 elevation post-pneumonia as possible indicator of ongoing NETosis and the HIST2H2BB decrease as indicator of prior histone protein release. Both responses were antagonized by amoxicillin.

Six proteins were elevated by pneumonia and not influenced to significant extent by amoxicillin (MVP, CACYBP, STK26, TOLLIP, HNRNPA3, COX7A2) (Fig. 3f). Of these, CACYBP was a calcyclin-binding protein involved in protein ubiquination and proteasomal degradation, STK26 a Golgi-associated serine-threonine kinase involved in the regulation of apoptosis, and COX7A2 a cytochrome-C oxidase polypeptide relevant for mitochondrial electron transport. Particularly notable was the increase of TOLLIP (toll-interacting protein) following pneumonia, which did not respond to amoxicillin (Fig. 3f). TOLLIP is a positive regulator of chronic leukocyte inflammation in response to lipopolysaccharide under conditions of atherosclerosis [25]. TOLLIP exacerbates ischemic injury through inflammatory responses after transient MCAO in mice [27]. Thus, pneumonia had proinflammatory consequences for neutrophil signaling, which were not reversed by the antibiotic.

### Neutrophils play a central role in brain injury associated with pneumonia

To elucidate the role of neutrophils in the pneumonia-associated brain injury, we depleted neutrophils by administering a monoclonal anti-Ly6G (1A8) antibody at 1 day before and 1 day after pneumonia (i.e., at 2 and 4 days post-MCAO) (time line see Suppl. Figure 7a). Neutrophil depletion at these subacute time points did not influence infarct volume, brain edema, IgG extravasation, and microvascular thrombus density at 2 dpp (that is, 5 days post-MCAO) in MCAO only mice, but reduced infarct volume, brain edema, IgG extravasation, and microvascular thrombus density in MCAO mice additionally exposed to pneumonia (Fig. 4a–d). Neurological deficits, ICAM1 expression on cerebral microvessels and the density of brain-infiltrated CD45^+^ leukocytes were not influenced by neutrophil depletion in MCAO only or MCAO/pneumonia mice (Suppl. Figure 7b; Suppl. Figure 8a, b). Brain hemorrhage formation was not influenced by neutrophil depletion, neither with respect to hemorrhage volume nor hemorrhage incidence (Suppl. Figure 8c, d). These findings indicated a central role of neutrophils in the histopathological injury associated with pneumonia.

### NET degradation by DNase-I fails to alleviate the neurological and histopathological consequences of pneumonia

Considering that neutrophils in peripheral blood carried a protein signature associated with neutrophil degranulation and NETosis, we next asked if the deleterious effects of pneumonia on ischemic injury were attributed to NETs, which are released by neutrophils upon severe cellular stress [6]. To clarify this question, we evaluated the effects of DNase-I administration starting 30 min before pneumonia (i.e., 3 days post-MCAO) (Suppl. Figure 7c), which as we have previously shown degrades NETs [44]. DNase-I did not influence neurological deficits, infarct volume, brain edema, IgG extravasation, and microvascular thrombosis at 2 dpp (5 days post-MCAO) in MCAO only or MCAO/pneumonia mice (Suppl. Figure 7d; Fig. 4e, f, h; Suppl. Figure 8e). Similar to neutrophil depletion, DNase-I did not alter microvascular ICAM1 expression and brain-infiltrated CD45^+^ leukocyte density (Suppl. Figure 8f, g). The lack of recovery-promoting effects was associated with the exacerbation of brain hemorrhage volume and incidence, which were increased by DNase-I in MCAO and MCAO/pneumonia mice (Fig. 4g; Suppl. Figure 8 h), in line with previous findings of our group for MCAO conditions not associated with pneumonia [51].

### NET formation blockade by gasdermin-D inhibitor prevents brain edema and microvascular thrombosis, but not neurological impairment

Since NET degradation by DNase-I induced brain hemorrhages, we asked if a more subtle intervention that does not entirely eliminate preexisting NETs may be able to reverse the consequences of pneumonia. Thus, we evaluated the effects of a NET formation blocker, the pharmacological gasdermin-D inhibitor LDC7559 (HY-111674; MedChemExpress, Monmouth Junction, NJ, U.S.A.) [51], which we administered 30 min before pneumonia (Suppl. Figure 7e). LDC7559 delivery reversed the increased levels of CitH3^+^ DNA, which is specific for NETs [44], in peripheral blood plasma (Suppl. Figure 9). Although LDC7559 administered at this delayed time point did not influence neurological deficits (Suppl. Figure 7f) and infarct volume (Fig. 4i), it reduced pneumonia-induced brain edema and microvascular thrombosis at 2 dpp (Fig. 4j, l). IgG extravasation, ICAM1 expression on cerebral microvessels and brain-infiltrated CD45^+^ leukocyte density were not significantly influenced by LDC7559 (Suppl. Figure 8i-k). Of note, NET formation blockade by LDC7559 did not increase brain hemorrhage volume or incidence (Fig. 4k; Suppl. Figure 8l). Hence, LDC57559 reversed the structural histopathological, but not functional neurological consequences of pneumonia in the subacute stroke phase.

### Myeloperoxidase inhibition reduces brain edema and microvascular thrombosis, but not neurological impairment

Once released by neutrophils, NETs form aggregates with neutrophil-derived proteins, namely myeloperoxidase (MPO) and histones including histone-H4, which induce cell injury under ischemia/reperfusion conditions [11, 42]. To clarify the contribution of MPO to pneumonia-associated brain injury, we administered the pharmacological inhibitor AZD4831 (HY-145581; MedChemExpress) [18] 2 h before pneumonia (Suppl. Figure 7g). Plasma MPO activity doubled upon pneumonia, and MPO inhibition by AZD4831 reversed it (Suppl. Figure 10). AZD4831 did not influence neurological deficits (Suppl. Figure 7h) and infarct volume (Fig. 4m) at 2 dpp, but reversed the pneumonia-induced BBB breakdown, indicated by brain edema and IgG extravasation (Fig. 4n, o), and reduced microvascular thrombosis (Fig. 4p). ICAM1 expression on cerebral microvessels, brain CD45^+^ leukocyte infiltrates and intracerebral hemoerrhages were not altered by AZD4831 (Suppl. Figure 8m–p). Thus, AZD4831 alleviated the structural, but not functional consequences of pneumonia.

### Histone blockade decreases infarct volume, brain edema, microvascular thrombosis, and neurological impairment

To neutralize histones, we administered the monoclonal anti-histone-H4/H2A antibody BWA3 (Creative Biolabs, Shirley, NY, USA) [4] 30 min before and 1 day after pneumonia (Suppl. Figure 7i). Histone blockade reduced neurological deficits (Suppl. Figure 7j) and infarct volume (Fig. 4q) of MCAO/pneumonia mice at 2 dpp, prevented the pneumonia-associated BBB breakdown (Fig. 4r, s) and reduced microvascular thrombosis (Fig. 4t). Microvascular ICAM1 expression, brain CD45^+^ leukocyte infiltrates, and intracerebral hemorrhages were not influenced by histone blockade (Suppl. Figure 8q–t). Taken together, histone blockade reversed the neurological and neuropathological consequences of pneumonia in the early stroke recovery phase.

### Pneumonia worsens long-term neurological and histopathological stroke outcome

**Fig. 5:**
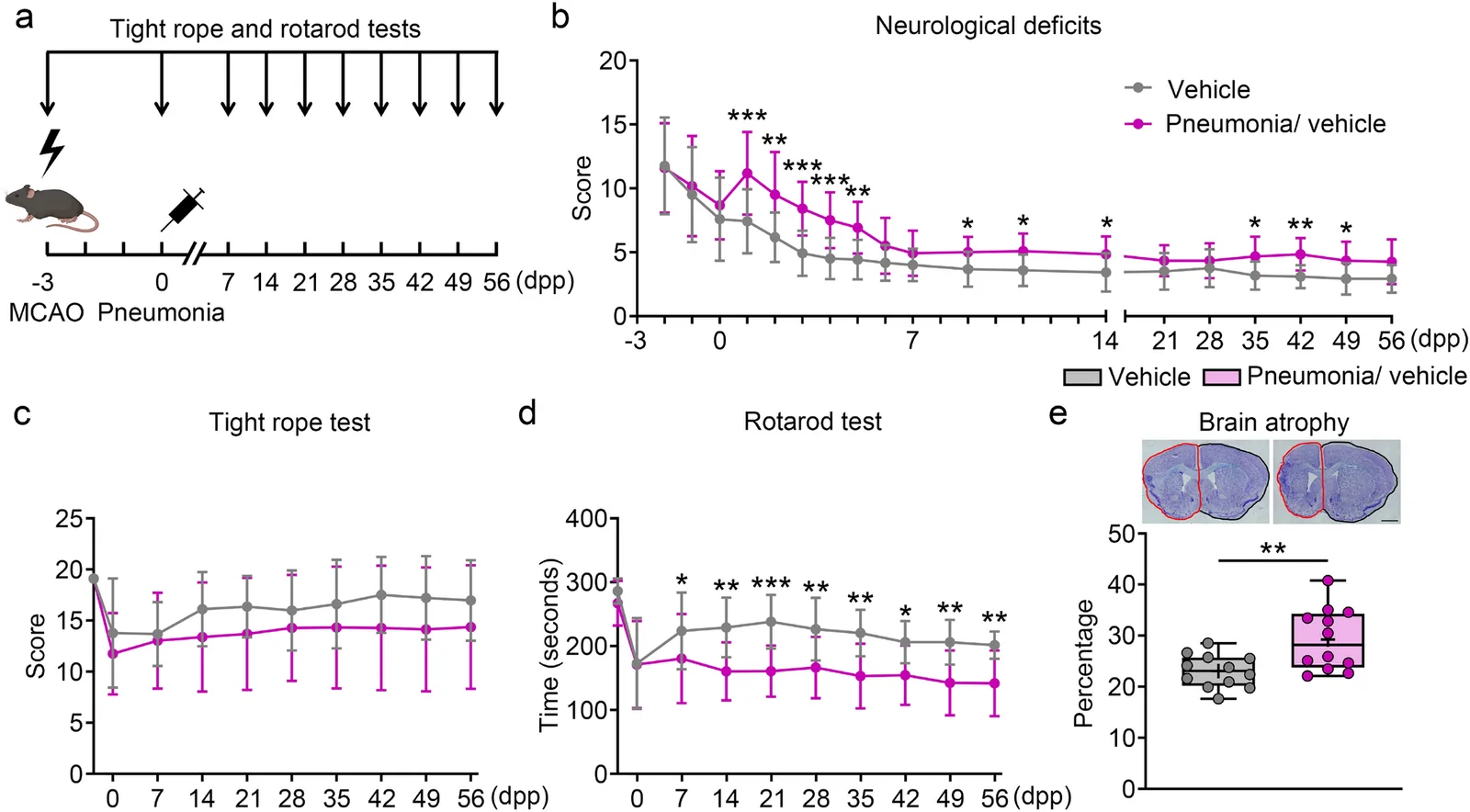
Pneumonia compromises long-term functional and structural stroke outcome. **a** Time line of animal experiments, **b–d** functional deficits evaluated using the neurological score, tight rope and RotaRod tests, and **e** brain atrophy of MCAO mice not exposed to pneumonia or MCAO mice exposed to bacterial pneumonia at 3 days post-MCAO, which were sacrificed at 56 dpp. Representative cresyl violet stainings are depicted. *p < 0.05, **p < 0.01, ***p < 0.001 (n = 12/group). Scale bars: 1 mm (in **e**). Mouse illustration in **a** created in BioRender.com

To elucidate the long-term consequences of pneumonia for functional and structural stroke outcome, we exposed MCAO mice to *S. pneumoniae* pneumonia at 3 days post-MCAO and examined neurological recovery over 56 dpp (time line see Fig. 5a). Pneumonia significantly exaggerated behavioral deficits assessed by the neurological score, tight rope and Rotarod tests, which did not fully recover over 56 dpp (Fig. 5b–d). Of note, brain atrophy at 56 dpp was significantly increased by pneumonia (Fig. 5e), indicating compromised structural brain remodeling in stroke/pneumonia mice.

### NET formation inhibition during pneumonia does not influence long-term stroke recovery

Considering its effects on BBB integrity and microvascular thrombus formation, we next asked if NET formation blockade using LDC7559 promotes long-term stroke recovery in MCAO/pneumonia mice. LDC7559 treatment on occasion of pneumonia (that is, 3 days after MCAO; time line in Suppl. Figure 11a) did not enhance functional and structural stroke outcome assessed by the neurological score, tight rope, RotaRod, open field and novel object recognition tests, brain atrophy, and microglial/macrophage density and activation over up to 56 dpp (Suppl. Figure 11b–l). In contrast, LDC7559 administered immediately after MCAO (time line in Suppl. Figure 12a) significantly reduced behavioral deficits in the neurological score and tight rope tests, increased spontaneous motor activity in open field tests, increased novel object discrimination in object recognition tests, reduced brain trophy, and reduced microglial/macrophage activation (Suppl. Figure 12b–l). Our results indicate that NET formation blockade was effective only at early stages as long as NET release has taken place.

### Extracellular histone neutralization during pneumonia induces long-term functional and structural stroke recovery

**Fig. 6:**
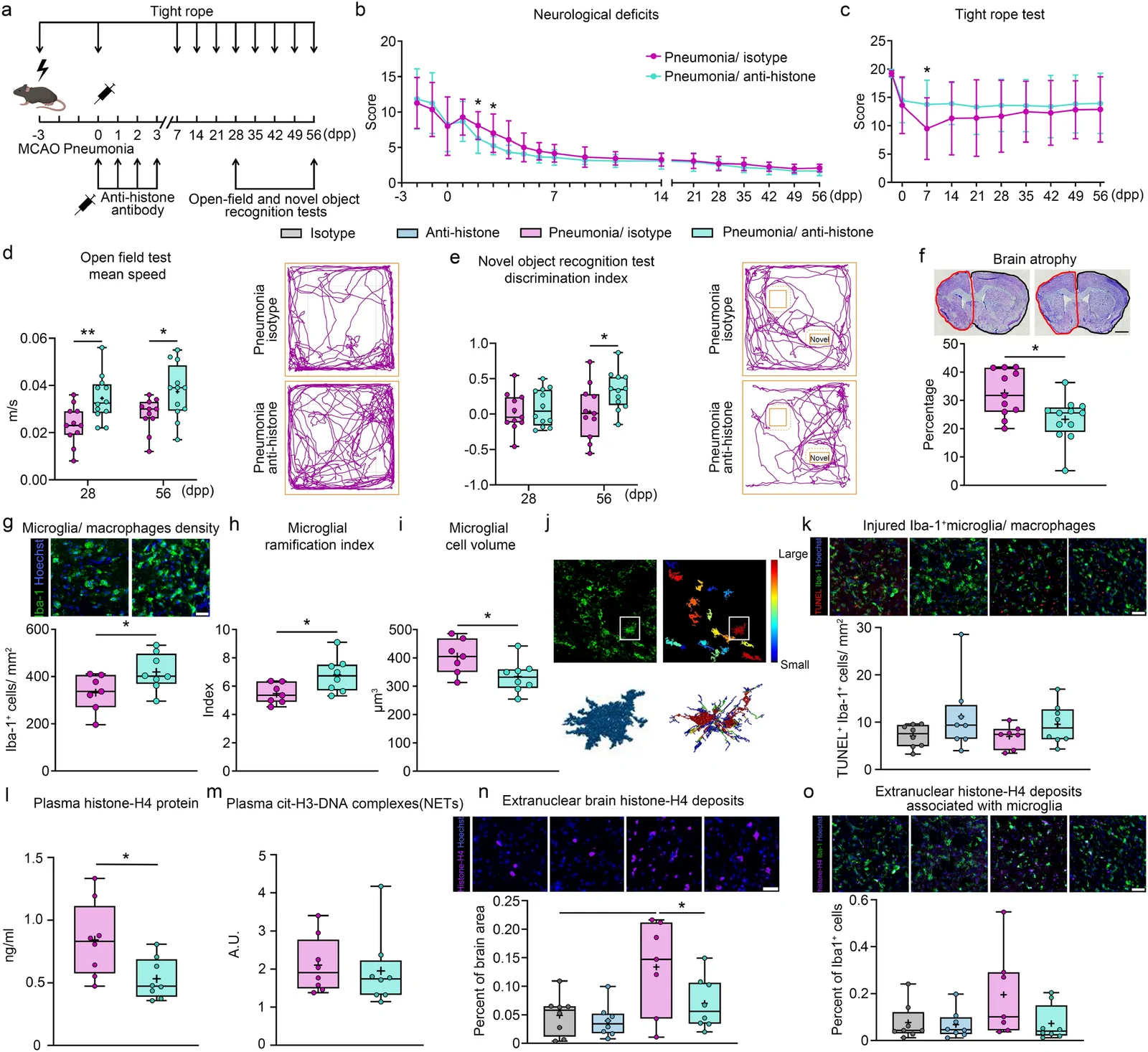
Extracellular histone neutralization promotes long-term neurological recovery and prevents brain atrophy in mice with stroke-associated pneumonia. **a** Time line of animal experiments, **b**, **c** functional deficits evaluated by the neurological score and tight rope tests, **d** spontaneous motor activity in open field tests (tracking paths of representative animals at 56 dpp are shown), **e** novel object recognition in object recognition tests (tracking paths of representative animals at 56 dpp are also shown), **f** brain atrophy, assessed by cresyl violet staining (representative images are depicted), **g–i** density and activation of Iba1^+^ microglia/macrophages in the ischemic striatum, assessed by cell counting and morphology analysis following segmentation, skeletonization and reconstruction (a representative cell segmentation and skeleton is shown in **j**), **k** irreversibly injured (that is, DNA-fragmented) Iba1^+^ microglia/macrophages in the ischemic striatum, assessed by Iba1/TUNEL doublelabeling, **l** extracellular histone-H4 protein and **m** citrullinated histone-H3 (Cit-H3)-DNA complexes (that is, NETs) in the plasma, **n** extranuclear histone-H4 deposits and **o** extranuclear histone-H4 protein associated with Iba1^+^ microglia/macrophages in the ischemic striatum of MCAO mice exposed to bacterial pneumonia, which were treated with isotype IgG or neutralizing anti-histone-H4/H2A antibody (BWA3; 20 µg i.p.) 30 min before pneumonia, followed by animal sacrifice at 56 dpp (behavioral and histochemical studies in **b–f**) or 2 dpp (histochemical and morphology studies in **g–o**). Representative cresyl violet and immunohistochemistry images are shown. *p < 0.05, **p < 0.01 (n = 11–12 mice/group (in **b–f**); n = 7–8 mice/group (in **g–o**)). Scale bar: 1 mm (in **f**); 50 μm (in **k**, **o**); 30 μm (in **g**, **n**). Mouse illustration in **a** created in BioRender.com

Considering its effects on the functional and structural sequelae of pneumonia in the early stroke recovery phase, we finally asked if extracellular histone-H4/H2A neutralization using the BWA3 antibody enhances long-term stroke recovery post-pneumonia. Histone-H4/H2A blockade during pneumonia (that is, 3 days after the stroke; time line in Fig. 6a) reduced behavioral deficits assessed by the neurological score and tight rope tests (Fig. 6b, c), increased spontaneous motor activity in open field tests (Fig. 6d) and novel object recognition in object recognition tests (Fig. 6e), and reduced brain atrophy (Fig. 6f) evolving over up to 56 dpp. Histone blockade also increased microglial/macrophage density (Fig. 6g) and reduced microglial/macrophage activation assessed by ramification index and cell volume (Fig. 6h–j), yet without influencing microglial/macrophage cell death, assessed by Iba1/TUNEL colabeling (Fig. 6k). Histone-H4/H2A blockade reduced plasma extracellular histone-H4 protein (Fig. 6l), but not plasma citrullinated histone-H3 (Cit-H3)-DNA complexes (that is, NETs) (Fig. 6m). Thus, NETosis was not influenced by histone blockade. Histone blockade significantly reduced extranuclear histone-H4 protein deposits in the ischemic brain (Fig. 6n), including (albeit not to statistically significant extent) extranuclear histone-H4 deposits associated with Iba1^+^ microglia/macrophages (Fig. 6o). Rectal temperature, body weight, bacterial load in BALF, lung inflammation assessed by HE and PAS staining, as well as plasma IgA and IgG were not influenced by histone blockade (Suppl. Figure 13a–i). Taken together, histone blockade persistently improved long-term neurological and histopathological outcome when administered 3 days after stroke in MCAO/pneumonia mice.

## Discussion

Using complementary analyses in ischemic stroke patients and in mice subjected to middle cerebral artery occlusion, we demonstrate that stroke-associated pneumonia markedly worsens stroke outcome. This process is neutrophil-associated. It involves BBB breakdown, cerebral microvascular thrombosis, and progressive brain atrophy. Antibiotic treatment with amoxicillin only partially ameliorated the pneumonia-associated neurological deficits, confirming that infection control alone is insufficient to prevent secondary brain damage. Of note, extracellular histone neutralization by anti-histone-H4/H2A antibody improved the long-term neurological and neuropathological consequences of pneumonia. On the contrary, NET degradation, NET formation blockade or MPO inhibition did not provide lasting functional and structural benefits.

Microvascular permeability and thrombosis are well-established features of bacterial pneumonia and sepsis [3, 55]. Infection-remote microvascular thrombosis is known from severe acute respiratory syndrome-corona virus-2 infection (SARS-CoV-2), particularly in the heart and less commonly the brain [2, 39]. Microvascular thrombosis in remote organs is rare in *S. pneumoniae* pneumonia, except for hemolytic uremic syndrome, a fulminant condition characterized by severe thrombocytopenia and hemolytic anemia [29]. To our knowledge, BBB breakdown and cerebral microvascular thrombosis have not yet been reported in *S. pneumoniae* pneumonia, neither in stroke-associated pneumonia nor pneumonia in other contexts.

Neutrophils are key components of dynamic capillary stalls that impede the reopening of reperfused blood vessels, promoting microvascular thrombosis and ischemic brain damage [13, 38]. Given that neutrophils travel from the infected lungs to cerebral vessels, we hypothesized neutrophils mediate the exacerbated brain injury observed in stroke/pneumonia mice. Indeed, anti-Ly6G antibody-mediated neutrophil depletion reversed the detrimental effects of pneumonia on BBB breakdown and microvascular thrombosis, and reduced ischemic brain injury. However, neutrophils are central for bacterial clearance [23], and post-stroke immunodepression hampers adaptive immunity [44]. Thus, neutrophil depletion is not a clinically viable option in stroke patients. Since antibiotic treatment with amoxicillin failed to reduce brain neutrophil infiltration in our stroke/pneumonia model, we sought alternative strategies to mitigate the harmful consequences of pneumonia on the ischemic brain.

Proteome analysis showed that stroke-associated pneumonia induces a degranulation, platelet activation, and NETosis signature in mouse neutrophils, which was partially reversed by amoxicillin. Microvascular thrombi are heavily enriched with neutrophil-derived DNA NETs [15, 26], which promote platelet aggregation and thrombus formation [10, 26], contributing to reperfusion resistance and ischemic damage after stroke [10, 54]. Given this, we investigated whether removing NETs could reverse the harmful effects of pneumonia. Contrary to our hypothesis, NET depletion by DNase-I did not alleviate the pneumonia-associated deterioration of stroke damage. Instead, it increased hemorrhagic transformation of brain infarcts in line with previous studies of our group for delayed DNase-I delivery in MCAO conditions not associated with pneumonia [51]. These data suggest that NETs play a crucial role in the sealing of ischemic blood vessels. Three randomized controlled trials currently assess the effects of intravenously administered DNase-I in ischemic stroke patients (clinicaltrials.gov/study/NCT05203224, NCT05880524, NCT04785066). These studies should carefully monitor signs of hemorrhagic transformation after DNase-I treatment. In our study, DNase-I tendentially reduced microvascular thrombosis in MCAO/pneumonia mice; this effect failed to reach statistical significance. The lack of more pronounced effects may be attributed to in the increase of brain hemorrhages promoting platelet aggregation.

To inhibit NET formation, we utilized the pharmacological gasdermin-D blocker LDC7559. Unlike DNase-I, which eliminates all circulating DNA (including DNA released by necrotic cells) [6, 11], LDC7559 more selectively reduces NETs in ischemic stroke mice [44]. LDC7559 did not increase hemorrhagic transformation, but reduced BBB leakage and microvascular thrombosis in stroke/pneumonia mice. The different effects of DNase-I and LDC7559 on microvascular integrity and brain hemorrhages can be explained by their diverse modes of action. Unlike DNase-I, LDC7559 does not influence DNA release by necrotic cells nor DNA deposits which have already been formed before drug administration. Of note, NET formation blockade by LDC7559 enhanced long-term neurological recovery and reduced brain atrophy only, when administered immediately after reperfusion. Whether the microglial/macrophage deactivation induced by LDC7559 results from the anti-pyroptotic effect of the drug or whether it simply reflects reduced brain injury, remains to be examined. Our findings confirm that post-ischemic NET formation is a rapid process [44] requiring early intervention to improve stroke outcome [10].

Our next target, the NET-bound peroxidase MPO, activates a large number of proteases, including matrix metalloproteases (MMPs), via its product HOCl/OCl^–^ through conversion of cysteine into thiol residues [33, 47]. In case of MMPs, this conversion facilitates the cleavage of pro-MMPs to MMPs including MMP-9 [24, 47], which contributes to BBB breakdown and brain injury after tissue-plasminogen activator-induced thrombolysis in embolic mouse stroke models [46]. Due to its diffusible nature, HOCl/OCl^–^ was a strong candidate to mediate BBB breakdown and thrombosis in infection-remote vascular beds [33]. Although MPO deactivation by AZD4831 antagonized the pneumonia-associated BBB breakdown and microvascular thrombosis, it did not restore neurological deficits, when administered during pneumonia. Pharmacological MPO inhibition has previously reduced neurological deficits, brain edema, and ischemic injury when initiated immediately after MCAO in mice [52].

In subsequent studies, extracellular histone proteins emerged as the only targets via which functional and structural stroke outcome could successfully be enhanced. Thus, antibody-mediated neutralization of extracellular histone-H4/H2A proteins during pneumonia improved long-term neurological recovery in stroke/pneumonia mice and prevented BBB breakdown, microvascular thrombosis, microglial activation, and brain atrophy. Extracellular histones, most notably histone-H4, are released by neutrophils during NETosis similar to parenchymal cells during necrosis, which induce smooth muscle cell damage in mouse atherosclerosis models [42]. Injured smooth muscle cells were found to attract neutrophils, promoting the ejection of NETs containing nuclear proteins including histone-H4 [42]. This reiterated process destabilizes atherosclerotic plaques, whereas histone-H4 neutralization stabilizes atherosclerotic plaques [42]. Histone-H4 proteins have previously been studied in experimental sepsis models in mice and baboons, in which they reduced mortality and renal dysfunction, respectively [50]. Administration of histone proteins was shown to increase neurological deficits and infarct volume in MCAO mice [9]. In the latter study, anti-histone antibody treatment decreased infarct volume, but not neurological deficits. Once in the extracellular space, histones act as damage-associated molecular patterns that evoke cell injury through toll-like receptor (TLR) and complement activation [32, 40]. TLR activation induces cytokine/chemokine release via MyD88, nuclear factor-κB and NLR family pyrin domain containing protein-3 (NLRP3), which drives tissue inflammatory responses [40]. Histone neutralization was thus expected to have overriding effects on stroke outcome, which our data indeed showed. That histone neutralization protects the brain against the consequences of pneumonia is new. Our findings advocate histone neutralization as a potential treatment in ischemic stroke patients. Small-molecule inhibitors, inhibitory peptides, and neutralizing antibodies for histone-H4 are currently in development for clinical use [32]. Their administration might enhance neurological outcome in stroke-associated pneumonia patients.

## Supplementary Information

Below is the link to the electronic supplementary material.

Supplementary file1 (DOCX 64944 KB)

## Data Availability

The mass spectrometry data have been deposited to the ProteomeXchange Consortium via the PRIDE partner repository with the dataset identifier PXD053004 and will be publicly made accessible upon publication. Other data are available from the corresponding author upon reasonable request.
