# Supplementary material for: Histone neutralization protects the ischemic brain against the consequences of stroke-associated pneumonia
Source: Acta Neuropathol. 2026 Oct 9;152(1):50. doi: 10.1007/s00401-026-03097-0 (PMC13652931; doi:10.1007/s00401-026-03097-0)
Supplement: Supplementary file 1 [file 401_2026_3097_MOESM1_ESM.docx]

**Legends to Supplementary figures:**

**Suppl. Fig. 1. Gating strategy used for analyzing blood leukocytes and leukocyte activation by flow cytometry.** In two sets of studies, **(a)** lymphoid and **(b)** myeloid cells were examined in mice exposed to middle cerebral artery occlusion (MCAO) followed by *S. pneumoniae* pneumonia.

**Suppl. Fig. 2. *S. pneumoniae* pneumonia induces post-ischemic hypothermia, which is attenuated by amoxicillin alongside the improvement of lung infection. (a)** Rectal temperature, **(b)** body weight, **(c-e)** bacterial load in the bronchoalveolar fluid (BALF), blood, and brain, **(f)** lung inflammation examined by HE staining and **(g)** lung mucus formation assessed by PAS/ HE staining, as well as **(h)** extranuclear histone-H4 deposits associated with Iba1^+^ microglia/ macrophages in the ischemic brain tissue (striatum) of mice subjected to transient 30 minutes intraluminal MCAO, which were exposed to pneumonia induced by intratracheal *S. pneumoniae* instillation (1x10^8^ CFUs) at 3 days post-MCAO, followed by vehicle or amoxicillin (15 mg/kg t.i.d., s.c.) treatment starting 3 hours thereafter. BALF, blood, brain and lung samples were taken at 2 days post-pneumonia (dpp; i.e., 5 days post-MCAO), which was the time point of animal sacrifice. *p<0.05, **p<0.01, ***p<0.001 compared with no pneumonia/ vehicle; ^#^p<0.05, ^##^p<0.01, ^###^p<0.001 compared with no pneumonia/ amoxicillin; ^+^p <0.05 compared with pneumonia/ vehicle (n=4 mice/group in **(a)**, n=7-11 mice/group in **(b)**, and n=8 mice/group in **(c-h)**). Scale bars: 200 μm (in **(f)**), 50 µm (in **(g, h)**).

**Suppl. Fig. 3. Bacterial pneumonia** **and amoxicillin treatment do not affect blood-brain barrier integrity, microvascular thrombus formation, or inflammatory responses in the contralateral hemisphere post-stroke. (a)** IgG extravasation, **(b)** ICAM-1 expression on microvessels, **(c)** CD45^+^ leukocyte density, **(d)** microvascular thrombus density, and **(e-g)** density and activation of Iba1^+^ microglia in the homologous contralateral striatum, assessed by cell counting and morphology analysis following segmentation, skeletonization, and reconstruction, of 30 minutes MCAO mice, which were not exposed to pneumonia or exposed to *S. pneumoniae* pneumonia at 3 days post-MCAO, followed by vehicle or amoxicillin (15 mg/kg t.i.d., s.c.) treatment starting 3 hours later. Mice were sacrificed at 2 dpp (i.e., 5 days post-MCAO). Representative immunohistochemistry images are shown. No significant differences were noted between groups (n=7-11 mice/group). Scale bars: 1 mm (in **(a)**); 50 μm (in **(b, c, e)**); 30 μm (in **(d)**).

**Suppl. Fig. 4. Bacterial pneumonia increases neurological deficits, brain edema, brain inflammatory responses and microvascular thrombus formation in a severe ischemic stroke model. (a)** Time line of animal experiments, **(b)** neurological deficits assessed by a comprehensive behavioral score, **(c)** infarct volume, and **(d)** brain edema evaluated by cresyl violet staining, **(e)** IgG extravasation in ischemic brain tissue, **(f)** ICAM1 expression on ischemic microvessels, **(g)** brain infiltrated CD45^+^ leukocytes, **(h)** microvascular thrombus density in ischemic brain tissue, and **(i-k)** density and activation of Iba1^+^ microglia/ macrophages in the ischemic striatum (i.e., the core of the middle cerebral artery vascular territory), assessed by cell counting and morphology analysis following cellular segmentation, skeletonization, and reconstruction (a representative cell segmentation and skeleton is shown in **(l)**), of 60 minutes transient MCAO mice, which were not exposed to pneumonia or exposed to bacterial pneumonia at 3 days post-MCAO, followed by animal sacrifice 2 days later (i.e., 5 days post-MCAO). Representative cresyl violet and immunohistochemistry images are depicted. *p<0.05, **p<0.01 (n= 7-8 mice/ group). Scale bars: 1 mm (in **(c-e)**); 50 μm (in **(f, g, i)**); 30 μm (in **(h)**). Mouse illustration in **(a)** created in BioRender.com.

**Suppl. Fig. 5. Stroke-associated pneumonia increases the number and activation of neutrophils in peripheral blood, which is reversed by amoxicillin.** Number of CD45^+^ leukocytes, Ly6G^+^ neutrophils, Ly6G^+^ CXCR2^+^ CD62L^high^ non-activated neutrophils, Ly6G^+^ CXCR2^+^ CD62L^int^ activated neutrophils, CD115^+^ monocytes, CD115^+^ Ly6C^neg^ patrolling monocytes, CD115^+^ Ly6C^int^ intermediate monocytes, CD115^+^ Ly6C^high^ inflammatory monocytes, CD115^-^ CD11b^+^ CD62L^+^ macrophages, CD3ε^+^ T cells, CD4^+^ T cells, CD4^+^ CD69^+^ activated T cells, CD8^+^ T cells, CD8^+^ CD69^+^ activated T cells, B220^+^ B cells, and NK1.1^+^ NK cells in peripheral blood assessed by flow cytometry of 30 minutes MCAO mice, which were not exposed to pneumonia or exposed to bacterial pneumonia at 3 days post-MCAO, followed by vehicle or amoxicillin treatment (15 mg/kg t.i.d., s.c.) starting 3 hours later. Animals were sacrificed at 2 dpp. Note that besides neutrophils, CD8^+^ activated T cells were also increased by pneumonia. Amoxicillin reduced CD8^+^ T cell numbers in peripheral blood of pneumonic mice. *p<0.05, **p<0.01 (n=6-8 mice/ group).

**Suppl. Fig. 6. Amoxicillin does not influence plasma IgA, plasma IgG, histone-H4 and citrullinated histone-H3-associated DNA levels in stroke-associated pneumonia. (a)** Plasma IgA and **(b)** plasma IgG, which are markers of adaptive immune function, **(c)** extracellular histone-H4 protein and **(d)** citrullinated histone-H3 (Cit-H3)-associated DNA (that is, neutrophil-derived extracellular DNA trap, NET) levels in the plasma of 30 minutes MCAO mice, which were exposed to bacterial pneumonia at 3 days post-MCAO, followed by vehicle or amoxicillin (15 mg/kg t.i.d., s.c.) treatment starting 3 hours later. Plasma samples were taken at 2 dpp (i.e., 5 days post-MCAO). No significant differences were noted between groups (n=8 mice/group).

**Suppl. Fig. 7. Effects of antibody-mediated neutrophil depletion, neutrophil extracellular DNA trap (NET) degradation, NET formation blockade, myeloperoxidase (MPO) inhibition, and histone neutralization on post-ischemic neurological deficits. (a, c, e, g, i)** Time line of animal experiments and **(b, d, f, h, j)** neurological deficits evaluated by a comprehensive behavioral score of 30 minutes MCAO mice not exposed to pneumonia or MCAO mice exposed to bacterial pneumonia at 3 days post-MCAO, which were treated with **(a, b)** isotype antibody or anti-Ly6G antibody (1A8; 200 µg i.p.), which depletes neutrophils (*15*), 1 day before and 1 day after pneumonia (i.e., at 2 and 4 days post-MCAO), **(c, d)** vehicle or DNase-I (10 µg i.v. and 50 µg i.p. 30 min before pneumonia, followed by 50 µg b.i.d.), **(e, f)** vehicle or the gasdermin-D inhibitor LDC7559 (10 mg/kg i.p.), which prevents NET formation (*19*), 30 min before pneumonia and 1 day after pneumonia, **(g, h)** vehicle or the MPO inhibitor AZD4831 (10 µmol/kg via oral gavage) 2 hours before and 1 day after pneumonia, or **(i, j)** isotype IgG or neutralizing anti-histone antibody (BWA3; 20 µg i.p.) 30 min before and 1 day after pneumonia, followed by animal sacrifice at 2 dpp (that is, 5 days post-MCAO). *p<0.05, **p<0.01, ***p<0.001 (n=6-10 mice/ group).

**Suppl. Fig. 8. Effects of neutrophil depletion, NET degradation, NET formation blockade, MPO inhibition, and histone neutralization on post-ischemic cerebral microvascular ICAM1 abundance, brain leukocyte infiltrates, and brain hemorrhage formation.** Cerebral microvascular ICAM1 abundance, **(b, g, k, n, r)** brain-infiltrated CD45+ leukocyte density, **(c, o, s)** brain hemorrhage volume, **(d, h, l, p, t)** hemorrhage incidence, and **(e, i)** IgG extravasation in the previously ischemic brain tissue of 30 minutes MCAO mice not exposed to pneumonia or MCAO mice exposed to bacterial pneumonia at 3 days post-MCAO, which were treated with **(a-d)** isotype antibody or anti-Ly6G antibody (1A8; 200 µg i.p.), which depletes neutrophils (*15*), 1 day before and 1 day after pneumonia (i.e., at 2 and 4 days post-MCAO), **(e-h)** vehicle or DNase-I (10 µg i.v. and 50 µg i.p. 30 min before pneumonia, followed by 50 µg b.i.d.), **(i-l)** vehicle or the gasdermin-D inhibitor LDC7559 (10 mg/kg i.p.), which prevents NET formation (*19*), 30 min before pneumonia and 1 day after pneumonia, **(m-p)** vehicle or the MPO inhibitor AZD4831 (10 µmol/kg via oral gavage) 2 hours before and 1 day after pneumonia, or **(q-t)** isotype IgG or neutralizing anti-histone-H4/H2A antibody (BWA3; 20 µg i.p.) 30 min before and 1 day after pneumonia, followed by animal sacrifice at 2 dpp (that is, 5 days post-MCAO). Representative immunohistochemistry images are shown. *p<0.05, **p<0.01 (n=6-10 mice/ group). Scale bars: 50 µm (in **(a, b**, **f, g**, **j, k**, **m, n, q, r)**; 50 µm (in **(c, o, s)**; 1 mm (in **(e, i)**).

**Suppl. Fig. 9. Effects of NET formation blockade by LDC7559 on DNA-associated citrullinated histone-H3 levels in the blood of mice with stroke-associated pneumonia.** Citrullinated histone-H3 (Cit-H3) protein associated with DNA assessed by capture ELISA in plasma samples obtained 48 hours post-pneumonia (120 hours post-MCAO) from 30 minutes MCAO mice not exposed to pneumonia or MCAO mice exposed to bacterial pneumonia at 3 days post-MCAO, which were treated with vehicle or the gasdermin-D inhibitor and NET formation blocker LDC7559 (10 mg/kg i.p.) 30 min before pneumonia. *p<0.05 (n=7-10 mice/ group).

**Suppl. Fig. 10. Effects of MPO inhibitor AZD4831 on MPO activity in the blood of stroke-associated pneumonia mice.** MPO activity determined using a colorimetric activity assay in plasma samples obtained 24 hours post-pneumonia (96 hours post-MCAO) from 30 minutes MCAO mice not exposed to pneumonia or MCAO mice exposed to bacterial pneumonia at 3 days post-MCAO, which were treated with vehicle or the MPO inhibitor AZD4831 (10 µmol/kg via oral gavage) 2 hours before pneumonia. ***p<0.001 (n=6 mice/ group).

**Suppl. Fig. 11. NET formation blockade does not influence post-ischemic long-term neurological outcome and brain atrophy, when initiated on occasion of pneumonia. (a)** Time line of animal experiments. Motor-coordination deficits evaluated using **(b)** the neurological score, **(c)** tight rope tests, and **(d)** RotaRod tests, **(e)** spontaneous motor activity assessed by open field tests (tracking paths of representative animals are also shown), **(f)** novel object recognition assessed by object recognition tests (tracking paths of representative animals are also shown), **(g)** whole brain atrophy and **(h)** striatum atrophy, assessed by cresyl violet staining, and **(i-k)** density and activation of Iba1^+^ microglia/ macrophages in the ischemic striatum, assessed by cell counting and morphology analysis following cellular segmentation, skeletonization, and reconstruction (a representative segmentation and skeleton is shown in **(l)**), of 30 minutes MCAO mice not exposed to pneumonia or MCAO mice exposed to bacterial pneumonia at 3 days post-MCAO, which were treated with vehicle or the NET formation blocker LDC7559 (10 mg/kg i.p.) starting on occasion of pneumonia, followed by animal sacrifice at 56 dpp (behavioral and histochemical studies in **(b-h)**) or animal sacrifice at 2 dpp (microglia/ macrophage density and morphology studies in **(i-l)**). Representative images in **(g, h, l)** are depicted. No significant differences were noted between groups (n=11-12 mice/ group). Scale bars: 1 mm (in **(g, h)**); 30 μm (in **(i)**).

**Suppl. Fig. 12. NET formation blockade by LDC7559 enhances long-term neurological recovery, reduces microglial/ macrophage activation, and reduces striatum atrophy, when initiated immediately after the stroke. (a)** Time line of animal experiments. Motor-coordination deficits evaluated using **(b)** the neurological score, **(c)** tight rope tests, and **(d)** RotaRod tests, **(e)** spontaneous motor activity assessed by open field tests (tracking paths of representative animals are also shown), **(f)** novel object recognition assessed by object recognition tests (tracking paths of representative animals are also shown), **(g)** whole brain atrophy and **(h)** striatum atrophy, assessed by cresyl violet staining (samples of representative animals are also shown), and **(i-k)** density and activation of Iba1^+^ microglia/ macrophages in the ischemic striatum, assessed by cell counting and morphology analysis following cellular segmentation, skeletonization, and reconstruction (a representative segmentation and skeleton is shown in **(l)**), of 30 minutes MCAO mice not exposed to pneumonia or MCAO mice exposed to bacterial pneumonia at 3 days post-MCAO, which were treated with vehicle or the NET formation blocker LDC7559 (10 mg/kg i.p.) immediately after MCAO, followed by animal sacrifice at 56 dpp (behavioral and histochemical studies in **(b-h)**) or animal sacrifice at 2 dpp (microglia/ macrophage density and morphology studies in **(i-l)**). Representative images in **(g, h, k)** are shown. *p<0.05 (n=16 mice/ group). Scale bars: 1 mm (in **(g, h)**); 30 μm (in **(i)**).

**Suppl. Fig. 13. Extracellular histone neutralization does not influence bacterial load in the broncholaveolar fluid or lung inflammation in stroke-associated pneumonia. (a)** Rectal temperature, **(b)** body weight, **(c-e)** bacterial load in the bronchoalveolar fluid (BALF), blood, and brain, **(f)** lung inflammation examined by HE staining, **(g)** lung mucus formation assessed by PAS staining, as well as **(h)** plasma IgA and **(i)** plasma IgG in 30 minutes MCAO mice, which were exposed to bacterial pneumonia at 3 days post-MCAO and treated with isotype IgG or neutralizing anti-histone-H4/H2A antibody (BWA3; 20 µg i.p.) starting 30 min before pneumonia. BALF, blood, brain and lung samples were taken at 2 dpp (i.e., 5 days post-MCAO), which was the time point of animal sacrifice. No significant differences were noted between groups (n=8 mice/group). Scale bars: 200 μm (in **(f)**), 50 µm (in **(g)**).

**Suppl. Tables:**

**Suppl. Table 1. Antibodies used for flow cytometry in ischemic stroke patients**

| Antigen | Conjugate | Host/isotype | Clone | Supplier |
| --- | --- | --- | --- | --- |
| Human CD45 | BV785 | Mouse IgG1, kappa | HI30 | BioLegend |
| Human CD66b | Fluorescein isothiocyanate (FITC) | Mouse IgG1 | 80H3 | Beckman Coulter |
| Human CD15 | BV605 | Mouse IgG1, kappa | W6D3 | BioLegend |
| Human CD62L | Phycoerythrin (PE) | Mouse IgG1 | DREG56 | Beckman Coulter |

**Suppl. Table 2. Antibodies used for flow cytometry in MCAO mice**

| Antigen | Conjugate | Host/isotype | Clone | Supplier |
| --- | --- | --- | --- | --- |
| Mouse CD45 | Pacific blue | Rat IgG2b, kappa | 30F11 | BioLegend |
| Mouse CD45 | BV 605 | Rat IgG2b, kappa | 30F11 | BioLegend |
| Mouse Ly6G | PE | Rat IgG2a, kappa | 1A8 | BioLegend |
| Mouse CXCR2  (CD182) | APC/Cyanine7 | Rat IgG2a, kappa | SA044G4 | BioLegend |
| Mouse CD62L | eFluor 450 | Rat IgG2a, kappa | MEL-14 | eBioscience |
| Mouse Ly6C | FITC | Rat IgM, kappa | AL21 | BD Biosciences |
| Mouse CD11b | Allophycocyanin (APC) | Rat IgG2b, kappa | M1/70 | eBioscience |
| Mouse CD115 | PE-Cy7 | Rat IgG2a, kappa | AFS98 | eBioscience |
| Mouse CD3ε | Alexa Fluor 647 | Hamster IgG | 145-2C11 | BioLegend |
| Mouse CD4 | BV 605 | Rat IgG2a, kappa | RM4-5 | BD Biosciences |
| Mouse CD8 | BV 786 | Rat IgG2a, kappa | 53-6.7 | BD Biosciences |
| Mouse B220 | PE | Rat IgG2a, kappa | RA3-6B2 | BD Biosciences |
| Mouse NK-1.1 | FITC | Rat IgG2a, kappa | PK136 | BD Biosciences |
| Mouse CD69 | PE-Cy7 | Hamster IgG | H1.2F3 | BioLegend |

**Suppl. Table 3. Animals and animal dropouts at various stages of the study.**

| Animal set | Group | Initial number | Before pneumonia | | After pneumonia | | Surviving animals | Overall mortality rate (%) |
| --- | --- | --- | --- | --- | --- | --- | --- | --- |
|  |  |  | Drop out number | Mortality rate (%) | Drop out number | Mortality rate (%) |  |  |
| Amoxicillin treatment (histochemistry) | Vehicle | 7 | 0 | 0 | 0 | 0 | 7 | 0 |
|  | Amoxicillin | 7 | 0 | 0 | 0 | 0 | 7 | 0 |
|  | Pneumonia/ vehicle | 14 | 2 | 14.29% | 1 | 8.33% | 11 | 21.43% |
|  | Pneumonia/ amoxicillin | 14 | 3 | 21.43% | 0 | 0 | 11 | 21.43% |
| Amoxicillin treatment  (flow cytometry) | Vehicle | 9 | 1 | 11.11% | 0 | 0.00% | 8 | 11.11% |
|  | Amoxicillin | 9 | 0 | 0.00% | 1 | 11.11% | 8 | 11.11% |
|  | Pneumonia/ vehicle | 9 | 0 | 0.00% | 2 | 22.22% | 7 | 22.22% |
|  | Pneumonia/ amoxicillin | 8 | 0 | 0.00% | 0 | 0.00% | 8 | 0.00% |
| Neutrophil depletion  (histochemistry) | Isotype | 7 | 0 | 0.00% | 1 | 14.29% | 6 | 14.29% |
|  | Ly6G | 7 | 0 | 0.00% | 0 | 0.00% | 7 | 0.00% |
|  | Pneumonia/ Isotype | 10 | 0 | 0.00% | 3 | 30.00% | 7 | 30.00% |
|  | Pneumonia/ Ly6G | 7 | 0 | 0.00% | 0 | 0.00% | 7 | 0.00% |
| DNase-I treatment (histochemistry) | Vehicle | 6 | 0 | 0.00% | 0 | 0.00% | 6 | 0.00% |
|  | DNase-I | 9 | 1 | 11.11% | 0 | 0.00% | 8 | 11.11% |
|  | Pneumonia/ Vehicle | 6 | 0 | 0.00% | 0 | 0.00% | 6 | 0.00% |
|  | Pneumonia/ DNase-I | 8 | 0 | 0.00% | 1 | 12.50% | 7 | 12.50% |
| LDC7559 treatment (histochemistry) | Vehicle | 11 | 1 | 9.09% | 0 | 0.00% | 10 | 9.09% |
|  | LDC7559 | 9 | 1 | 11.11% | 0 | 0.00% | 8 | 11.11% |
|  | Pneumonia/ Vehicle | 11 | 0 | 0.00% | 0 | 0.00% | 11 | 0.00% |
|  | Pneumonia/ LDC7559 | 13 | 1 | 7.69% | 0 | 0.00% | 12 | 7.69% |
| AZD4831 treatment (histochemistry) | DMSO | 9 | 1 | 11.11% | 1 | 12.50% | 7 | 22.22% |
|  | AZD4831 | 9 | 1 | 11.11% | 0 | 0.00% | 8 | 11.11% |
|  | Pneumonia/ DMSO | 9 | 1 | 11.11% | 0 | 0.00% | 8 | 11.11% |
|  | Pneumonia/ AZD4831 | 9 | 1 | 11.11% | 0 | 0.00% | 8 | 11.11% |
| Anti-histone treatment (histochemistry) | Isotype | 9 | 1 | 11.11% | 0 | 0.00% | 8 | 11.11% |
|  | Anti-histone | 9 | 1 | 11.11% | 0 | 0.00% | 8 | 11.11% |
|  | Pneumonia/ Isotype | 9 | 1 | 11.11% | 1 | 12.50% | 7 | 22.22% |
|  | Pneumonia/ Anti-histone | 9 | 0 | 0.00% | 1 | 11.11% | 8 | 11.11% |
| Pneumonia induction  (long-term study) | Vehicle | 15 | 2 | 13.33% | 1 | 7.69% | 12 | 20.00% |
|  | Pneumonia/ Vehicle | 15 | 1 | 6.67% | 2 | 14.29% | 12 | 20.00% |
| Anti-histone  treatment  (long-term study) | Pneumonia/ Isotype | 15 | 2 | 13.33% | 2 | 15.38% | 11 | 26.67% |
|  | Pneumonia/ Anti-histone | 15 | 1 | 6.67% | 2 | 14.29% | 12 | 20.00% |
| Delayed LDC7559 treatment  (long-term study) | Pneumonia/ Vehicle | 13 | 1 | 7.69% | 1 | 8.33% | 11 | 15.38% |
|  | Pneumonia/ LDC7559 | 13 | 2 | 15.38% | 0 | 0.00% | 11 | 15.38% |
| Immediate LDC7559 treatment  (long-term study) | Pneumonia/ Vehicle | 18 | 1 | 5.56% | 1 | 5.88% | 16 | 11.11% |
|  | Pneumonia/ LDC7559 | 18 | 1 | 5.56% | 1 | 5.88% | 16 | 11.11% |

All animals were exposed to 30 minutes MCAO. In the 60 minutes MCAO study, no dropouts were noted.
